# Visible light-driven benign synthesis of benzoxazine–sulfur copolymers: high-performance materials for electrochemical applications

**DOI:** 10.1039/d6sc04441g

**Published:** 2026-08-03

**Authors:** Shivani Yadav, Saad Zafar, Bimlesh Lochab

**Affiliations:** a Materials Chemistry Laboratory, Department of Chemistry, School of Natural Sciences, Shiv Nadar Institution of Eminence Delhi NCR India bimlesh.lochab@snu.edu.in

## Abstract

Inverse vulcanization is an effective strategy for transforming abundant elemental sulfur into functional polymeric materials with tunable physicochemical properties for advanced applications. However, conventional methods typically require elevated processing temperatures, which are often accompanied by the evolution of toxic H_2_S, and frequently yield highly crosslinked, poorly soluble networks that restrict material processability. Herein, we report a visible-light-mediated room-temperature inverse vulcanization approach for the bulk copolymerization of the benzoxazine monomer with elemental sulfur in the absence of catalysts or solvents under ambient conditions. Irradiation in the 400–500 nm range enables controlled photochemical activation of S_8_, initiating radical ring-opening copolymerization (rROP). Compared with thermal and catalyst-assisted routes, the light-driven process suppresses H_2_S evolution, improves solubility, and affords copolymers with distinct structural features and ∼3-fold lower thiol content. Comprehensive spectroscopic, thermal, and GPC analyses verify efficient sulfur incorporation (∼50 wt%) in the photo-triggered copolymerization, resulting in organo-sulfur networks with comparatively lower branching density, consistent with enhanced spatial and temporal regulation of polymer growth. Spin-trapping experiments in conjunction with EPR spectroscopy reveal the generation of sulfur-centered radicals under irradiation, while thiol quantification supports a controlled reaction pathway. Comparative evaluation of copolymers synthesized *via* different routes demonstrates that the light-mediated material exhibits superior electrochemical performance, delivering a specific capacitance of 551 ± 9 F g^−1^ at 0.5 A g^−1^ and retaining 97% capacitance after 2000 cycles. This visible-light-mediated inverse vulcanization strategy offers a sustainable and energy-efficient route to sulfur-rich, solution-processable copolymers with reasonable potential for fabrication on heat-sensitive substrates for advanced energy storage applications.

## Abstract 2

Visible-light-mediated inverse vulcanization provides a sustainable, low-energy route to sulfur-rich benzoxazine copolymers, reducing toxic H__2__S emissions while enabling solution processing on heat-sensitive substrates for advanced energy storage.

## Graphical abstract

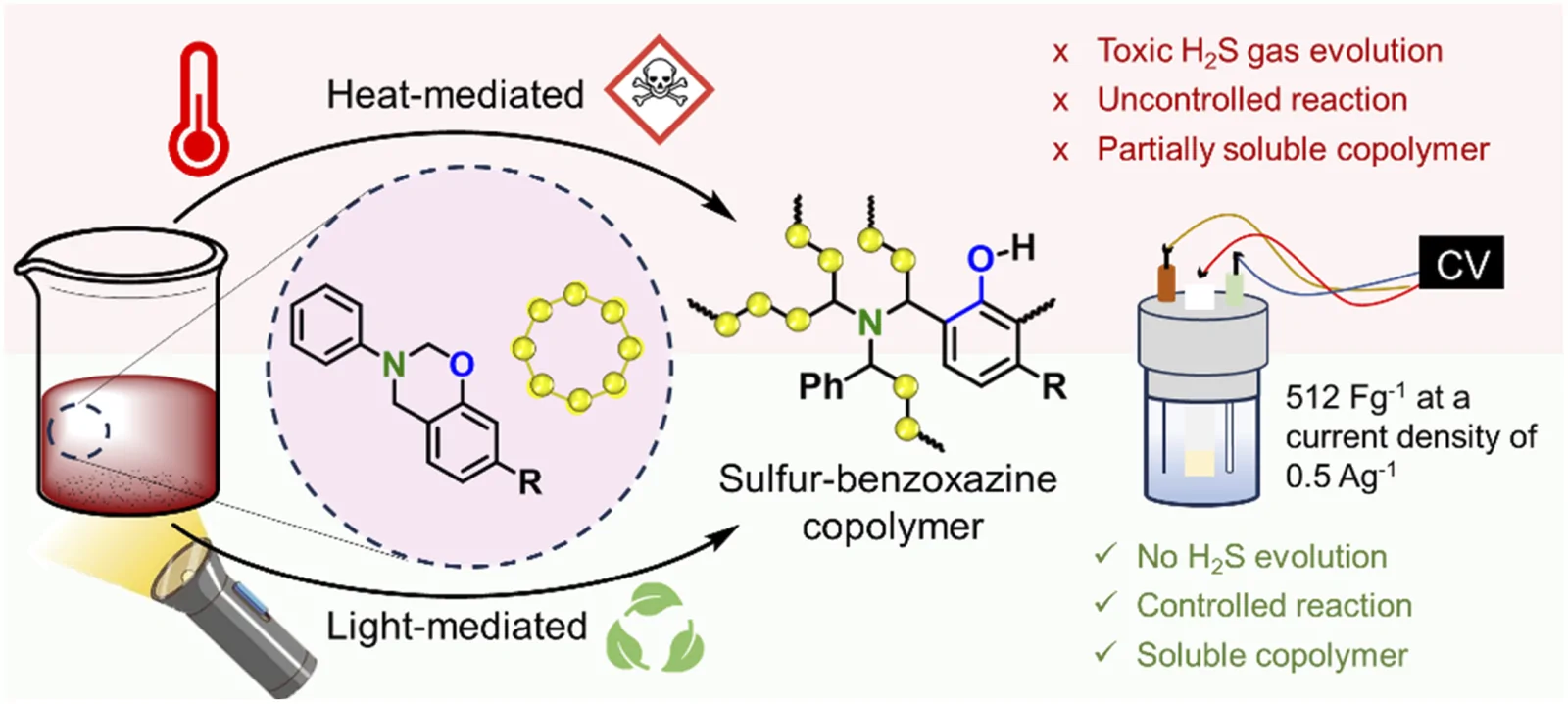

## Introduction

Polybenzoxazines (PBZs) have emerged as a versatile and technologically advanced class of thermosetting resins offering significant advantages over conventional phenolic resins. Benzoxazine (BZ) monomers are typically synthesized in a single-step *via* atom-economical Mannich-type condensation of phenols, amines, and aldehydes, and the monomers exhibit excellent shelf stability under ambient conditions. Upon heating, these monomers undergo thermally induced cationic ring-opening polymerization through a zwitterionic phenoxide–iminium intermediate, producing highly crosslinked PBZ networks.^1^

The inherent modularity and simplicity of BZ synthesis enable broad structural tunability and facile incorporation of functional groups. This structural diversity provides good control over thermomechanical properties, the glass-transition temperature (*T*_g_), substrate compatibility, hydrophobicity, and surface energy. As a result, PBZs display exceptional thermal and mechanical stability, high char yield, flame retardance, resistance to chemicals and water, low dielectric constants, and low surface free energy.^2–6^ These attributes have expanded PBZ applications from conventional uses—such as composites, coatings, and adhesives—to emerging areas including re(de)bondable adhesives, electrochemical materials, water-purification media, and gas-capture materials relevant to aerospace, energy, and environmental technologies. BZ monomers offer excellent co-reactivity ensuring new copolymers with attractive structure–property tunability and diversity.

Among the diverse comonomers explored, elemental sulfur (S_8_) is particularly attractive due to its ability to participate in multiple reactions with BZ *via* inverse vulcanization, enabling high sulfur incorporation. Sulfur is naturally abundant and economically attractive as a major by-product of petrochemical desulfurization.^7–9^ Foundational studies by Pyun^10^ and co-workers demonstrated that S_8_ copolymerizes with simple dienes to yield polymers with high refractive indices,^11,12^ intrinsic self-healing behavior,^13–17^ electrochemical activity,^18–21^ and antimicrobial properties.^22–25^ High sulfur-enriched polymers are stabilized against depolymerization of polysulfane by reaction with organic cross-linkers. Sulfur-rich copolymers have therefore gained substantial attention as electroactive components in supercapacitors and metal–sulfur batteries due to multielectron redox behavior, rapid redox kinetics, and high theoretical specific capacity of sulfur.^26–29^

Integrating sulfur into PBZ networks has emerged as an active research area because sulfur profoundly influences both polymerization kinetics and material performance. For example, elemental sulfur reduces the benzoxazine polymerization temperature from ≥200 °C to ∼120 °C,^30^ by promoting early formation of Schiff-base and phenolic groups during oxazine ring-opening. Rapid formation of poly(benzoxazine-*r*-sulfur) from BZ and sulfur at 180 °C (≤10 min) has also been reported, along with demonstration of their utility as cathodes in metal–sulfur batteries.^31^ One-pot syntheses of benzoxazine–sulfur–ionone terpolymers further broadened the electrochemical materials landscape.^32^

Additional versatility arises from co-reactions with allyl-functional benzoxazines,^33,34^ enabling diverse network architectures. Introduction of dynamic S–S linkages impart reprocessability, recyclability, and self-healing typical hallmarks of vitrimeric PBZs.^35–37^ Liu *et al.*^38^ showed that sulfur radical transfer and coupling (SRTC) reactions above 159 °C generate sulfur–benzoxazine copolymers and the proposed possible mechanism is *via* radical-mediated pathways. Even the presence of sulfur in the monomer/catalyst reduced the ROP temperature. For example, thioamide-catalyzed benzoxazine polymerization at ∼45 °C, driven by tautomerism-induced transient thiol generation,^39^ and reductive cleavage of aliphatic^40^ or aromatic^41^ disulfide-bridged benzoxazines yield reactive thiols that subsequently form thiazolidine intermediates providing the diversity of sulfur-enabled low-temperature PBZ chemistries. Sodium polysulfide (Na_2_S_*x*_) allowed a facile strategy for room temperature knitting of sulfur in polybenzoxazine for MWIR-transparent optics,^42^ and adaptable networks with intrinsic self-healing and antimicrobial properties.^43^

Over the past, significant progress has been made; however, requirement of elevated curing temperature sulfur–BZs including other unsaturated monomers leading to partially or fully insoluble networks, and more critically, formation of hydrogen sulfide (H_2_S)—a highly flammable and acutely toxic gas^44,45^—raise concerns for industrial applications. Among other stimuli, light attracts more attention due to its advantages of facile operation, non-invasiveness, and exceptional spatial and temporal control. These challenges highlight the opportunity for developing milder, simpler, more controllable, and energy-efficient polymerization routes that minimize H_2_S liberation. Further experimental proof to gain mechanistic insights also remain elusive.

**Fig. 1:**
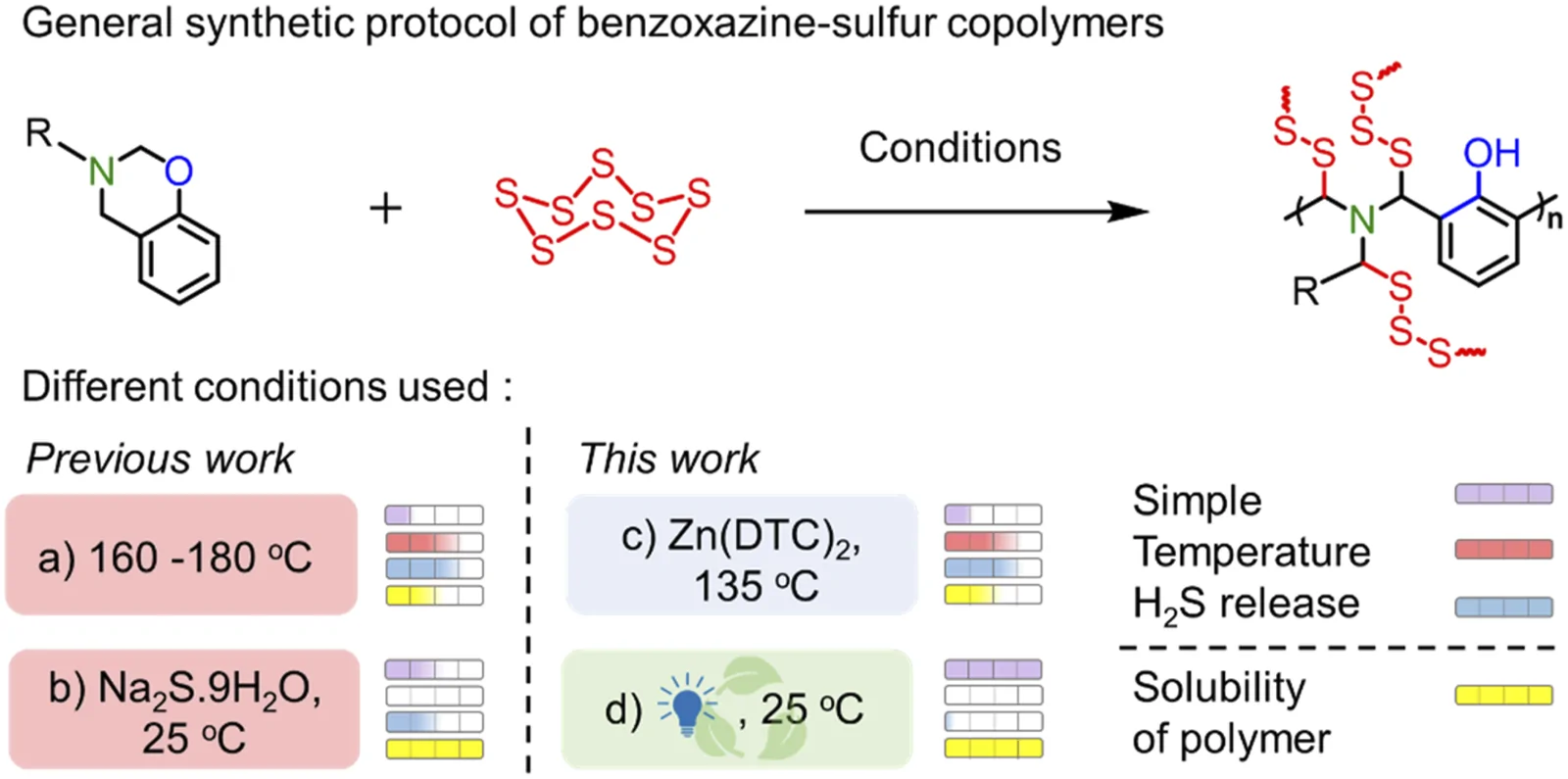
Valorization of elemental sulfur in polybenzoxazine for the synthesis of poly(benzoxazine-*r*-sulfur) copolymers.

We report here the preparation and investigation of photo-mediated inverse vulcanization in improving the reaction and safety in preparation of benzoxazine-based copolymers under ambient conditions, combining the advantages of both ring-opening polymerization and radical copolymerization. For comparison, sulfur–benzoxazine copolymers were also synthesized *via* the conventional high-temperature and catalytic route, as illustrated in Fig. 1. Visible-light irradiation (400–500 nm) initiates sulfur–benzoxazine coupling reactions through a solvent-free, catalyst-free, and energy-efficient process forming the copolymer. The light-governed radical ring-opening copolymerization (rROP) process not only eliminates H_2_S evolution from the reaction but also dramatically reduces the polymerization temperature to room temperature and the copolymer revealed excellent solution processability. Finally, we demonstrate the applicability of the prepared sulfur copolymers (contain high sulfur loading, ∼48 wt%) as cathodes for supercapacitors, establishing light-driven inverse vulcanization with enhanced electrochemical responsiveness and present a sustainable and tunable approach to next-generation sulfur–PBZ functional materials. This newly developed polymerization strategy enables better structural control over the temperature mediated copolymerization of sulfur and thus makes great advances in the field of sulfur copolymer synthesis.

## Results and discussion

### Synthesis of copolymers under thermal, catalytic, and photo-mediated conditions

**Fig. 2:**
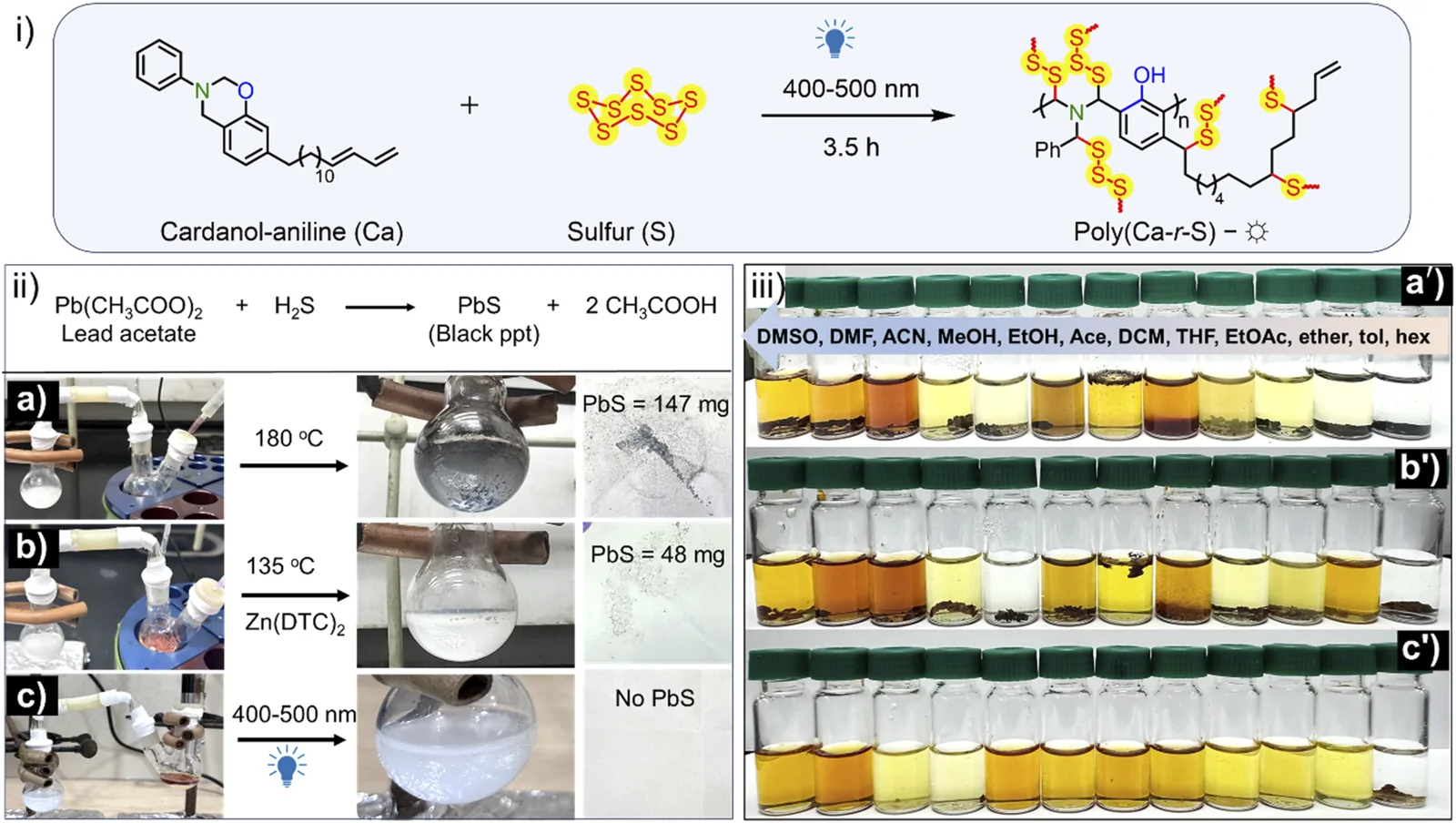
Synthesis of copolymers and solubility. (i) Schematic illustration of the photo-mediated ring-opening copolymerization between S_8_ and Ca. (ii) Comparative analysis of *in situ* H_2_S release during copolymerization at a Ca : S_8_ ratio corresponding to a 100 mg reaction scale, recorded after 1 h for panels (a and b) and after 3.5 h for panel (c). (iii) Solubility profiles of poly(Ca-*r*-S) in solvents of varying polarity (10 mg mL^−1^). Panels show copolymers obtained under (a and a′) thermal heating, (b and b′) heating in the presence of Zn(TDC)_2_, and (c and c′) light-mediated polymerization.

The synthesized cardanol-based BZ monomer (Ca, synthetic scheme and characterization is presented in Fig. S1) was copolymerized with S_8_ under three distinct reaction conditions namely (i) conventional high-temperature polymerization at 180 °C (copolymer denoted as poly(Ca-*r*-S) – Δ), (ii) a lower-temperature reaction at 135 °C in the presence of zinc dibutyldithiocarbamate, Zn(DTC)_2_ (copolymer denoted as poly(Ca-*r*-S) – ©) and (iii) a room-temperature, light-mediated polymerization at 25 °C (copolymer denoted as poly(Ca-*r*-S) – ☼), as illustrated in Fig. 2i and S2. For optimization of the photo-mediated copolymerization, initial screening experiments highlighted the critical role of irradiation wavelength in controlling monomer reactivity (Ca, S_8_, and their mixture) (Table S1). Control studies showed that irradiation of sulfur led to the formation of a yellow to “turmeric-like’’ solid (visual observation, Fig. S3a) whereas irradiation of Ca alone (400–500 nm) produced no detectable changes by ^1^H NMR spectroscopy (Fig. S3b), indicating occurrence of photochemical activation of S_8_. We hypothesize that visible light (400–500 nm) efficiently activates sulfur (see UV-visible spectra in Fig. S4), thereby enabling the reaction with the benzoxazine monomer at room temperature. In contrast, near-UV light (320–390 nm) resulted in incomplete conversion, likely due to insufficient spectral overlap and/or competing photochemical pathways that do not sustain effective chain transfer and propagation. No reaction was observed under dark conditions, confirming the photo-triggered nature of the process. Table S2 summarizes the reported benzoxazine–sulfur copolymerization pathways, and Table S3 represents inverse vulcanization approaches with alkene/alkyne monomers. Conventional sulfur–benzoxazine copolymerization approaches are typically constrained by several intrinsic challenges, including the reliance on high polymerization temperatures, the necessity for thermally stable monomers that do not undergo ring-opening polymerization at temperatures lower than those required for sulfur activation (generally ∼180 °C), and the tendency of the reaction to proceed in an uncontrolled manner affecting selectivity, often resulting in infusible networks affecting solution processability. Additional drawbacks include the generation of harmful H_2_S and limited control over material properties. Furthermore, benzoxazine monomers themselves conventionally require elevated temperatures for ring-opening polymerization, leading to highly crosslinked, insoluble polymer networks. To evaluate the reaction time required for efficient photo-induced copolymerization and influence of light intensity on the photoinduced inverse vulcanization process, *in situ* NMR kinetic studies of Ca were performed at different irradiation intensities, Fig. S5. The progression of oxazine ring-opening was monitored by following the characteristic Ar–CH_2_–N and O–CH_2_–N resonances. A clear dependence of monomer conversion on light intensity was observed, with the extent of ring-opening decreasing systematically as the irradiation intensity was reduced. At 100% light intensity, the Ar–CH_2_–N and O–CH_2_–N signals decreased by 98% and 95%, respectively. In contrast, the corresponding reductions were 44% and 30% at 50% intensity, and only 16% and 3% at 1% intensity, respectively. These results demonstrate that efficient copolymerization requires a minimum photon flux to effectively activate elemental sulfur and sustain the propagation process. At lower irradiation intensities, sulfur activation becomes significantly less efficient, resulting in reduced oxazine conversion and slower reaction kinetics. Consequently, efficient copolymerization under weak-light conditions may necessitate extended irradiation periods or increased light intensity to achieve sufficient sulfur activation and monomer conversion. The diminished conversion observed at lower light intensities correlates directly with the reduced extent of benzoxazine copolymerization (Fig. S6), supporting the role of photoinduced sulfur activation as the key rate-limiting step governing the reaction.

The observed trend is consistent with the photochemical requirement for sufficient excitation of elemental sulfur, supporting the premise that S_8_ ring-opening is the rate-determining step in the reaction and is mainly governed by light intensity. This interpretation is further corroborated by the work of Li *et al.*,^46^ which provided direct evidence for the generation of sulfur-centered radicals under UV irradiation through *in situ* EPR studies on pristine elemental sulfur. Additionally, Jia *et al.*^47^ demonstrated that the efficiency of photoinduced S_8_ ring-opening with unsaturated monomers is strongly wavelength-dependent, with no observable reaction occurring at wavelengths above 520 nm.

Evolution of toxic H_2_S during each reaction condition was assessed and quantified by passing the generated gas through a colorless lead acetate solution, Fig. 2ii. Formation of a characteristic black PbS precipitate was monitored over time. For the heat- and heat + catalyst-mediated reactions, the lead acetate solution darkened immediately, with the purely thermal reaction rapidly producing PbS, as confirmed from PXRD (Fig. S7) of the black precipitate. Gravimetric analysis showed that the heat + catalyst reaction generated ∼3-fold less PbS. In contrast, the light-mediated reaction produced no visible blackening, indicating an absence of detectable H_2_S evolution through the lead acetate test. In bulk thermal reactions, vigorous stirring and high temperature accelerate reaction kinetics, leading to rapid polymerization and the evolution of hazardous H_2_S gas. Moreover, elevated temperatures may contribute to autoacceleration effects due to the Trommsdorff–Norrish phenomenon. Conversely, the room-temperature bulk photo-induced pathway relies on diffusion of photo-generated radicals to drive chain propagation. During formation of poly(Ca-*r*-S), both sulfur and benzoxazine participate in multiple competing reactions, enabling the formation of cross-linked structures through intramolecular backbiting and intermolecular chain-transfer processes, which are generally favored at higher temperatures. Given the limited sensitivity of the lead acetate test, which serves primarily as a qualitative indicator of H_2_S, the evolution of H_2_S was further quantified using the more sensitive methylene blue assay.^48^ The concentrations of H_2_S generated under thermal, thermal + catalyst, and photo-mediated polymerization conditions were determined to be 25.9 ppm, 10.2 ppm, and 1.0 ppm, respectively (Fig. S8 and Table S4). These results reveal that photo-mediated copolymerization suppresses H_2_S generation by approximately 96% relative to thermal polymerization, demonstrating its effectiveness in suppressing undesirable toxic H_2_S emissions.

Solubility studies further distinguish the copolymers prepared under different conditions, Fig. 2iii. Heat-mediated copolymers exhibit low to partial solubility, displaying significant discoloration in both polar and non-polar solvents, whereas catalyst-assisted samples show slightly enhanced solubility. Remarkably, copolymers synthesized under 400–500 nm irradiation were highly soluble in all tested solvents except hexane. The suppression of H_2_S evolution and improved solubility of the photo-mediated materials indicate a distinct polymer architecture compared to that formed under thermal conditions.

To further quantify the dissolution, solubility tests were conducted in a series of solvents spanning a wide range of polarities. Across all copolymers, the solvent-dependent solubility trend followed the order: hexane < methanol < toluene < dichloromethane < acetonitrile. Importantly, within each solvent, the dissolution rate consistently followed the order: light-mediated > (heat + catalyst)-mediated > heat-mediated copolymers as shown in Fig. S9–S11. This enhanced network formation plausibly restricts polymer chain mobility and solvent ingression, resulting in reduced solubility. These additional experiments provided a coherent structure–property relationship linking synthesis conditions, network architecture, and solubility behavior.

### Comparative NMR analysis of copolymers prepared under different reaction conditions

To assess differences in the structural features of the copolymers obtained under various reaction conditions, a detailed comparative NMR analysis was conducted and plots are presented in Fig. S12. As expected, copolymerization of sulfur with Ca under thermal conditions was completed within 1 h, as evidenced by the complete disappearance of oxazine-ring resonances (O–CH_2_–N and N–CH_2_–Ar), alkylene-based unsaturated bonds, and vinylic, allylic, and benzylic (ArCH_2_–) signals. Additionally, new signals corresponding to imine and phenolic–OH protons appeared in the *δ* 8.6–11 ppm region, consistent with oxazine ring-opening reactions.

For comparison, photo-inverse vulcanization was performed for 1 h to obtain the copolymer poly(Ca-*r*-S) – ☼. Interestingly, the resulting ^1^H NMR spectrum showed changes only at the oxazine-ring protons, whose intensities were reduced relative to the monomer. No noticeable reactivity was observed at vinylic, benzylic, or aromatic positions, indicating that oxazine sites are more susceptible to photo-induced reactions with sulfur-derived radicals, forming organo-sulfur linkages. Extending the irradiation time to 3.5 h led to complete disappearance of the oxazine-ring signals, while allylic and benzylic resonances became broadened and slightly shifted, characteristic of polymer formation. Notably, terminal double-bond proton signals were retained. The imine and phenolic–OH signals in the light-mediated copolymer were significantly weaker than those observed under thermal conditions, suggesting their further involvement or consumption during copolymerization. Simultaneously, several new broad upfield resonances emerged between *δ* 2.3–4.0 ppm and *δ* 4.3–4.9 ppm, assigned to methylene and methine environments in –S–CH_2_– and –S–CH– fragments, respectively. These features are consistent with ring opening followed by sulfur incorporation, leading to phenolic/aryl–CH environments and aliphatic linkages typical of sulfur-coupled poly(benzoxazine-*r*-sulfur) architectures. These observations indicate that the mild nature of the light-mediated reaction affords improved control and selectivity compared to the thermally driven process. To confirm C–H one-bond correlations associated with these newly formed signals, ^1^H–^13^C HSQC spectra were acquired (Fig. S13). The observed cross-peaks between methine and methylene protons and their corresponding carbons were consistent with the assignments determined from 1D NMR spectra.

**Fig. 3:**
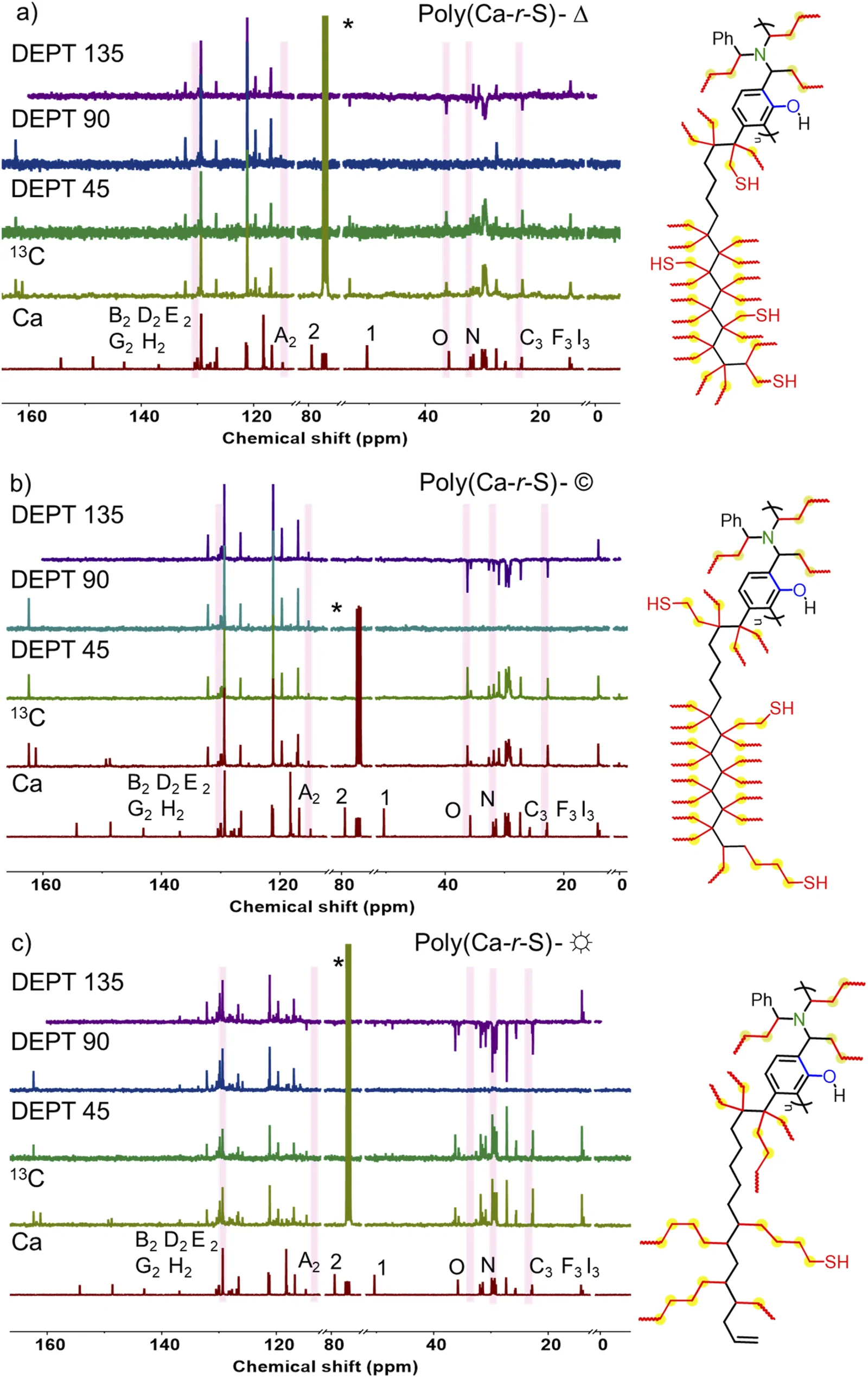
Stacked ^13^C and DEPT NMR spectra of the monomer with (a) poly(Ca-*r*-S) – Δ, (b) poly(Ca-*r*-S) – ©, and (c) poly(Ca-*r*-S) – ☼. Recorded in *CDCl_3_.

Further validation was obtained from ^13^C and DEPT NMR analyses (Fig. 3), which confirmed complete consumption of Ca and its co-reaction with sulfur under all conditions. Oxazine carbons (1 and 2), along with benzylic and adjacent heteroatom-bound carbons (N and O), were absent in all spectra. Terminal alkenyl carbons (A_2_ and B_2_) disappeared under thermal conditions, showed mono-substitution in the presence of catalyst (as verified by the CH signal in DEPT-90), and remained unchanged under light irradiation. Allylic (C_3_, F_3_, and I_3_) and alkene (D_2_, E_2_, G_2_, and H_2_) carbons were fully consumed under thermal and catalytic conditions but shifted in the light-mediated reaction, indicating extensive sulfur incorporation and the formation of predominantly quaternary carbon centers due to extensive substitution by sulfur. Methylene carbons exhibited substantial broadening, most pronounced in the heat-mediated samples, followed by the catalyst-assisted reaction, and least in the light-induced copolymer. Concurrently, new resonances between 24.0 and 50.0 ppm appeared, attributable to sulfur–carbon covalent linkages. Distinct phenolic–OH and imine carbons at 161.2 ppm and 162 ppm, respectively, confirmed the oxazine ring-opening and subsequent co-reaction with sulfur.

The significant chemical-shift changes across the alkylene, oxazine, and aromatic regions collectively support the formation of highly substituted and branched organo-polysulfane structures under thermal conditions, whereas the light-triggered pathway yields comparatively less branched architectures. Although inverse vulcanization *via* photopolymerization is a milder alternative to thermal polymerization, the former approach requires a longer reaction time (3.5 h) compared to the thermal approach (1 h).

Diffusion-ordered NMR spectroscopy (DOSY) further corroborated these results, showing a markedly lower diffusion coefficient for the pure heat-mediated copolymer compared to the Ca monomer, consistent with larger and more highly branched polymeric species (Fig. S14). The catalyst-assisted reaction exhibited the highest diffusion coefficient, suggesting formation of more compact and lower-molecular-weight products. In contrast, the room-temperature photo-induced copolymer displayed an intermediate diffusion coefficient, indicating that although reactivity occurred at fewer sites, the resulting polymer possessed appreciable molecular mass, demonstrating efficient propagation under mild photochemical conditions.

### Spectroscopic and thermal characterization of sulfur–benzoxazine copolymers

**Fig. 4:**
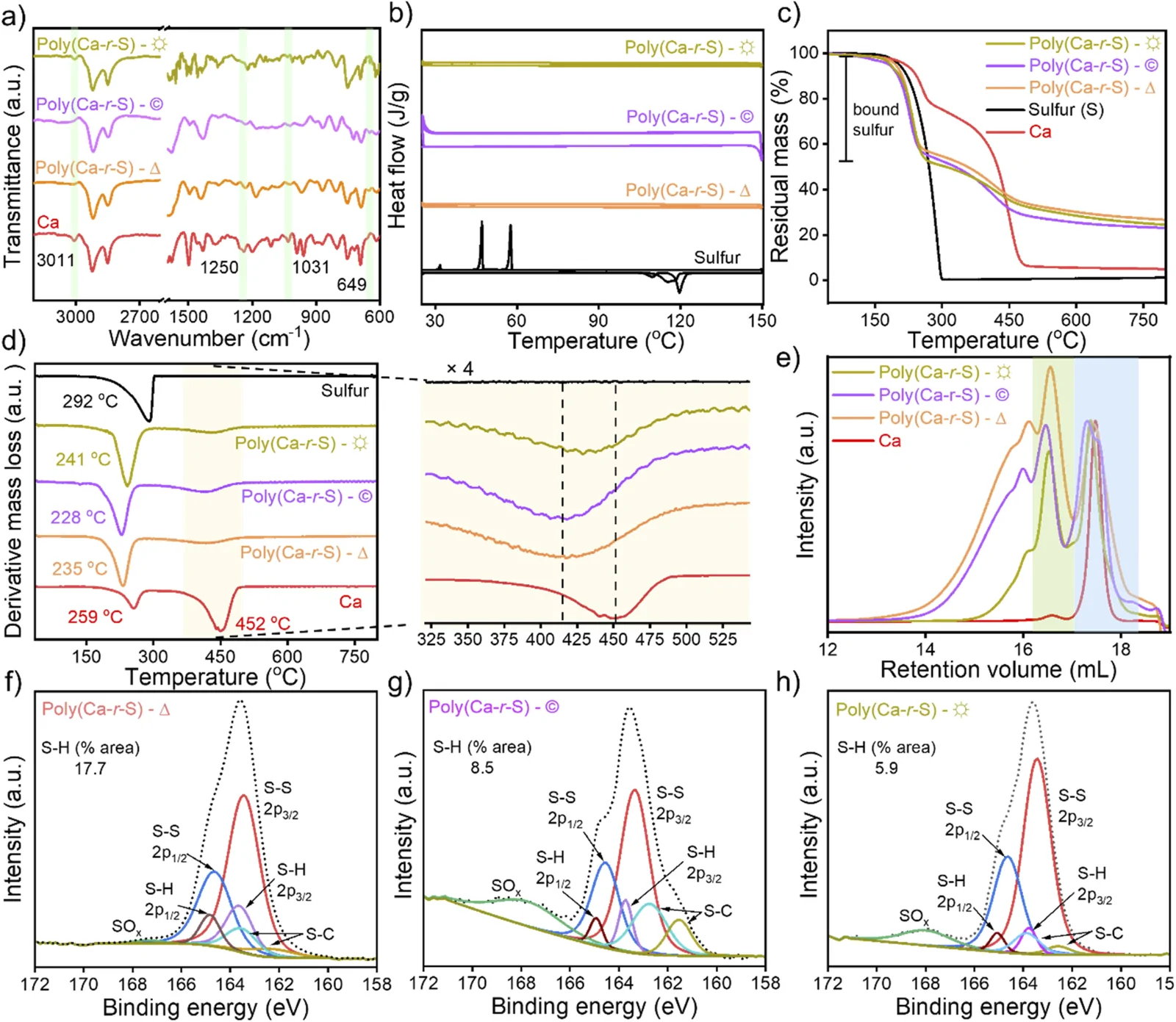
Structural and thermal characterization of the copolymers and corresponding monomers. (a) FTIR spectra. (b) Differential scanning calorimetry (DSC) profiles. (c) Thermogravimetric analysis (TGA) curves. (d) Differential thermogravimetry (DTG) plots. (e) Gel permeation chromatography (GPC) traces. (f–h) Deconvoluted S 2p XPS spectra.

FTIR analysis further confirmed the formation of organo-sulfur linkages and oxazine ring-opening. The characteristic C–S stretching vibration at 695 cm^−1^ appeared, while the oxazine-related stretching modes (C–O–C_asym_, C–O–C_sym_, and C–N–C at 1250, 1112, and 1031 cm^−1^, respectively) were absent (Fig. 4a). Additionally, the emergence of a broad phenolic O–H band near 3500 cm^−1^ also supports oxazine ring opening, complementing the NMR observations.

To verify complete sulfur consumption and to assess the efficiency of the photo-mediated copolymerization relative to the thermal processes, the copolymers were examined using multiple analytical techniques. Differential scanning calorimetry (DSC, Fig. 4b) showed no melting endotherm associated with crystalline sulfur, confirming its complete consumption and indicating the amorphous nature of the copolymers. Thermogravimetric analysis (TGA, Fig. 4c) revealed good agreement between the feed and bound sulfur contents (∼47–49%), demonstrating that even the mild photo-mediated strategy incorporates sulfur with an efficiency comparable to high-temperature reactions. Correlation of TG and DTG thermograms (Fig. 4c and d) confirmed the absence of thermal signatures of unreacted benzoxazine (*T*_max_ = 259 °C and 452 °C; char yield = 5%; residual mass at 800 °C) and elemental sulfur (*T*_max_ = 292 °C; char yield = 0%) monomers. Although the overall bound sulfur contents were similar, DTG illustrated a two-step mass-loss behavior with the *T*_max_ values varying across samples, typically ∼228–241 °C for sulfur-rich domains and ∼416–434 °C for organic-rich domains, indicating differences in sulfur–organic covalent connectivity. Notably, the photo-mediated copolymer displayed slightly higher *T*_max_ values (zoomed-in view, Fig. 4d), suggesting retention of the organic carbon framework and potentially longer grafted sulfur chains, consistent with NMR findings.

To further confirm complete sulfur consumption, Raman spectroscopy and powder X-ray diffraction (PXRD) were performed. Raman spectra (Fig. S15a) were featureless, and the PXRD patterns (Fig. S15b) exhibited broad amorphous diffractogram without Bragg reflections characteristic of orthorhombic S_8_ (23.2°, 25.9°, and 27.9° for the (2 2 2), (0 2 6), and (0 4 0) planes, respectively). These results confirm the absence of residual crystalline sulfur and the presence of sulfur atoms incorporated as amorphous organic–sulfur networks.

Gel permeation chromatography (GPC) provided additional support for successful copolymerization (Fig. 4e) and the results are presented in Table S5. Ca showed a retention time of 17 min. The appearance of new broad peaks at lower retention volumes (13, 13.8, and 15 min), along with pronounced peak broadening, confirms significant molecular-mass enhancement upon sulfur incorporation. Both the heat-mediated samples showed broad chromatographic peaks at lower retention volumes, indicating larger hydrodynamic radii and higher molecular masses. In contrast, the light-mediated copolymer exhibited a narrower peak indicating a relatively more controlled growth and the lowest hydrodynamic radii. GPC analysis using light scattering and viscosity detectors was performed, with representative plots shown in Fig. S16 and the results summarized in Table S6. As expected, the molecular weight followed a consistent trend—poly(Ca-*r*-S) – © < poly(Ca-*r*-S) – ☼ < poly(Ca-*r*-S) – Δ—irrespective of the detector employed. The viscometry data yielded very low “*a*” and “log *K*” values, precluding reliable interpretation of branching and rendering the results inconclusive. In contrast, light scattering measurements indicate a comparatively higher degree of branching in poly(Ca-*r*-S) – Δ.

Even though MALDI-ToF mass spectrometry (Fig. S17) data are complex, probably influenced by the inherent unsaturation of the side chain in cardanol and matrix-related ionization effects, but they still highlighted structural differences among the copolymers. For poly(Ca-*r*-S) – Δ, a major ion peak appeared at *m*/*z* 2106, likely corresponding to [C_32_H_59_NOS_51_]^+^. The catalyst-assisted copolymer exhibited a peak at *m*/*z* 1832 ([C_32_H_74_NOS_42_]^+^), while the light-mediated copolymer showed a peak at *m*/*z* 2074 ([C_32_H_59_NOS_50_]^+^). These findings suggest that all copolymers shared a backbone derived from the Ca monomer, with similar extent of sulfur incorporation irrespective of reaction conditions.

X-ray photoelectron spectroscopy (XPS) wide survey spectra (Fig. S18a–c) confirm the presence of carbon, oxygen, nitrogen and sulfur elements. Deconvolution of the C 1s (Fig. S18d–f) spectrum showed binding energies related to C–C/C=C and C–heteroatoms (O, N, and S) confirming successful copolymerization. The relative percentage associated with covalent linkages between carbon and sulfur is very high for heat-, followed by heat + catalyst, and least for photo-mediated driven copolymer. To determine the nature of sulfur incorporation and chemical-state distribution in the copolymers, the S 2p spectrum (Fig. 4f–h) was deconvoluted into four distinct peaks at 163.1, 164.0, 165.2, and 168.9 eV corresponding to S–C, S–H, S–S, and SO_*x*_, respectively and the results are tabulated in Table S7. Interestingly, the percentage of S–S bonding is major and that of S–H is minor in the light mediated copolymer, clearly confirming successful covalent interlacing of sulfur onto the polymer backbone with the long sulfur chains ascribed to catenation.

To evaluate the crosslink density of the resulting copolymers, temperature sweep rheological measurements were performed, Fig. S19a. The heat-mediated copolymer exhibited the highest crosslink density, Table S8, followed by the catalyst-mediated and light-mediated copolymers, respectively. Likewise, the complex viscosity (Fig. S19b) also followed a similar trend. These results further corroborate the distinct structural and properties imparted by the different copolymerization approaches.

### Radical intermediates and thiol participation in photo-driven benzoxazine–sulfur copolymerization

**Fig. 5:**
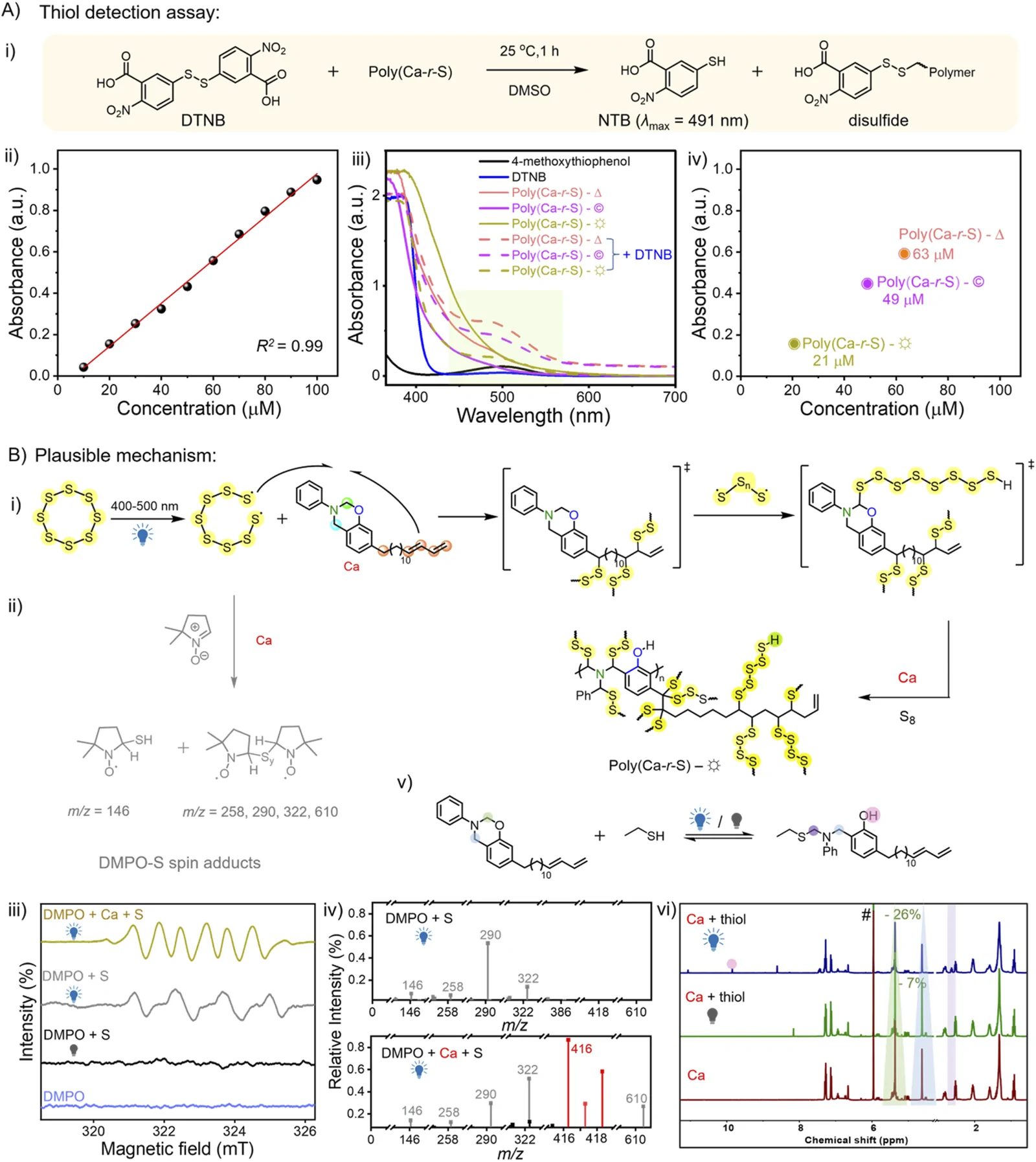
Mechanistic studies of the polymerization reaction. (A) Thiol quantification in copolymers: (i) exchange reaction of polymer-bound thiols with DTNB; (ii) calibration curve of DTNB and 4-methoxythiophenol standards using UV-vis spectroscopy; (iii) UV-vis absorbance spectra of the thiol-copolymer mixtures and respective controls; (iv) quantitative evaluation of thiol functionalities in the copolymers. (B) Radical ring-opening copolymerization: (i) proposed mechanism; (ii) control experiments confirming radical formation during the co-reaction; radical-trapping studies validating sulfur-radical generation as supported by (iii) EPR spectra and (iv) mass spectrometry. (v) Schematic representation for the effect of light and thiol on the oxazine ring-opening reaction; (vi) stacked NMR spectra (^#^1,3-benzoxazole used as an internal standard). All NMR spectra were recorded in CDCl_3_.

Given that thiols can undergo reversible addition to the benzoxazine ring and potentially influence the ring-opening pathway,^49^ the thiol content in the copolymers was quantified using Ellman's assay (DTNB), Fig. 5A-i. This method relies on a thiol–disulfide exchange reaction that generates the chromophoric TNB^−^ species, which is detectable by UV-vis spectroscopy.^50^ Fig. S20 shows the model reaction for the thiol-exchange reaction. Using a calibration curve, Fig. 5A-ii, iii, the thiol concentration was determined spectrophotometrically. Among the samples, poly(Ca-*r*-S) – Δ exhibited the highest thiol content (63 µM), followed by poly(Ca-*r*-S) – © (49 µM) and poly(Ca-*r*-S) – ☼ (20 µM), indicating significantly reduced thiol functionality under the photo-mediated condition, as plotted in Fig. 5A-iv.

A plausible light-mediated copolymerization mechanism of Ca and S_8_ is shown in Fig. 5B-i. To further elucidate the reactive intermediates responsible for copolymer formation, radical generation during photo-induced inverse vulcanization was probed through spin-trapping experiments using TEMPO and DMPO, Fig. 5B-ii and S21a–d. The resulting adducts were characterized by electron paramagnetic resonance (EPR) spectroscopy and mass spectrometry (MS), Fig. S21c and d, following established methodologies for radical detection.^51^ Upon irradiation of the Ca/S_8_/TEMPO, a dark-brown coloration developed, and HRMS revealed a new signal at *m*/*z* 406 (relative to Ca at *m*/*z* 416), consistent with the formation of a TEMPO–S adduct. EPR analysis showed a triplet signal (*g* = 2.008 and *a*_N_ = 15.67 G), characteristic of a TEMPO-based radical species, supporting the generation of sulfur-centered radicals under visible light. Since TEMPO is less effective at trapping highly reactive or short-lived sulfur radicals, DMPO was employed as a more efficient and sensitive spin-trapping agent.^52^ No EPR signal was observed for DMPO/S_8_ mixtures in the dark. Under irradiation, a distinct quartet (*a*_N_ = 9.4 G and *a*_H_ = 18.34 G) appeared, consistent with DMPO–SH formation (*m*/*z* 146) and a higher sulfur-containing adduct (*m*/*z* 258, 290, 322; *y* = 1–3 in DMPO–S_*y*_). In the presence of Ca, S_8_, and DMPO under irradiation, a characteristic sextet EPR pattern with hyperfine coupling constants (9.4, 16.7, and 24.3 G), consistent with the literature^52,53^ was observed, Fig. 5B-iii. Interestingly, even higher-order sulfur adducts (*m*/*z* 610, *y* ≈ 12) were obtained, Fig. 5B-iv, confirming generation of sulfur-centered radical species directly from photoactivated S_8_. The absence of DMPO–S_*y*_ signals in the dark excludes thermal photolysis and confirms the photochemical origin of these radicals.

The oxazine ring-opening copolymerization proceeded quantitatively under visible-light irradiation in the presence of elemental sulfur, while the resulting copolymer exhibits low thiol content. Control experiments using ethanethiol, performed with and without irradiation, Fig. 5B-v, vi, showed a 4-fold increase in ring opening under light (from 7% to 26%), yet conversion remained non-quantitative. This indicates that thiol-induced ring-opening is reversible and insufficient to account for the complete transformation observed under sulfur + light condition. Importantly, since only ∼26% monomer conversion can be attributed to the thiol-assisted, light-boosted copolymerization process, the remaining ∼74% conversion is attributed to elemental sulfur-mediated radical processes. This observation supports a mechanism in which sulfur-derived radicals are primarily responsible for driving the extensive ring-opening and copolymerization observed under photochemical conditions.

The results support a mechanism in which oxazine ring opening proceeds *via in situ*-generated thiol/thiolate species formed alongside sulfur-centered radicals under Ca/S_8_ irradiation, with their cooperative interaction enabling efficient copolymer formation and controlled reactivity under mild photo-mediated conditions. The above findings support a cooperative mechanism in which photoexcited sulfur generates sulfur-centered radicals alongside *in situ* thiol/thiolate species. The interplay between these reactive intermediates enables efficient oxazine ring opening and subsequent copolymer formation, providing a mechanistic basis for the high selectivity and controlled reactivity observed under mild photo-mediated conditions.

To assess whether the observed photochemical behavior is specific to benzoxazine or represents a more general phenomenon, additional monomers under identical irradiation conditions (400–500 nm) were reacted with sulfur as shown in Fig. S22 and S23. The progress of the photo-mediated inverse vulcanization of these monomers was monitored by NMR spectroscopy, as shown in Fig. S23. Analysis of the NMR spectra indicates that PHfa (containing only the oxazine ring) and C_sat_a (saturated benzoxazine analogue to Ca) exhibit conversions of ∼50% and ∼70%, respectively, which are significantly lower than that observed for Ca (Fig. S5d and S23b, d). These results highlight the influence of oxazine-ring reactivity on the overall conversion. In comparison to PHfa, which lacks alkyl substitution, the presence of a benzylic position in C_sat_a enhances the conversion by ∼20%. This reactivity is further increased by ∼28% in Ca, attributable to the presence of reactive carbon–carbon double bonds, highlighting their role in promoting photo-mediated inverse vulcanization. In contrast, the benzoxazine monomer Ca displayed a very high reactivity, than C_sat_a and PHfa, with elemental sulfur. The NMR spectra of DGEBA show nearly no changes upon reacting with sulfur, indicating its chemical inertness under the applied conditions. Additionally, 1,3-DIB exhibits a high monomer conversion of ∼90%, consistent with the well-established propensity of carbon–carbon double bonds to participate efficiently in (inverse) vulcanization processes.

### Exploration of copolymers for supercapacitor and room temperature processing

#### Electrochemical performance and charge-storage behavior of poly(Ca-*r*-S) copolymers

**Fig. 6:**
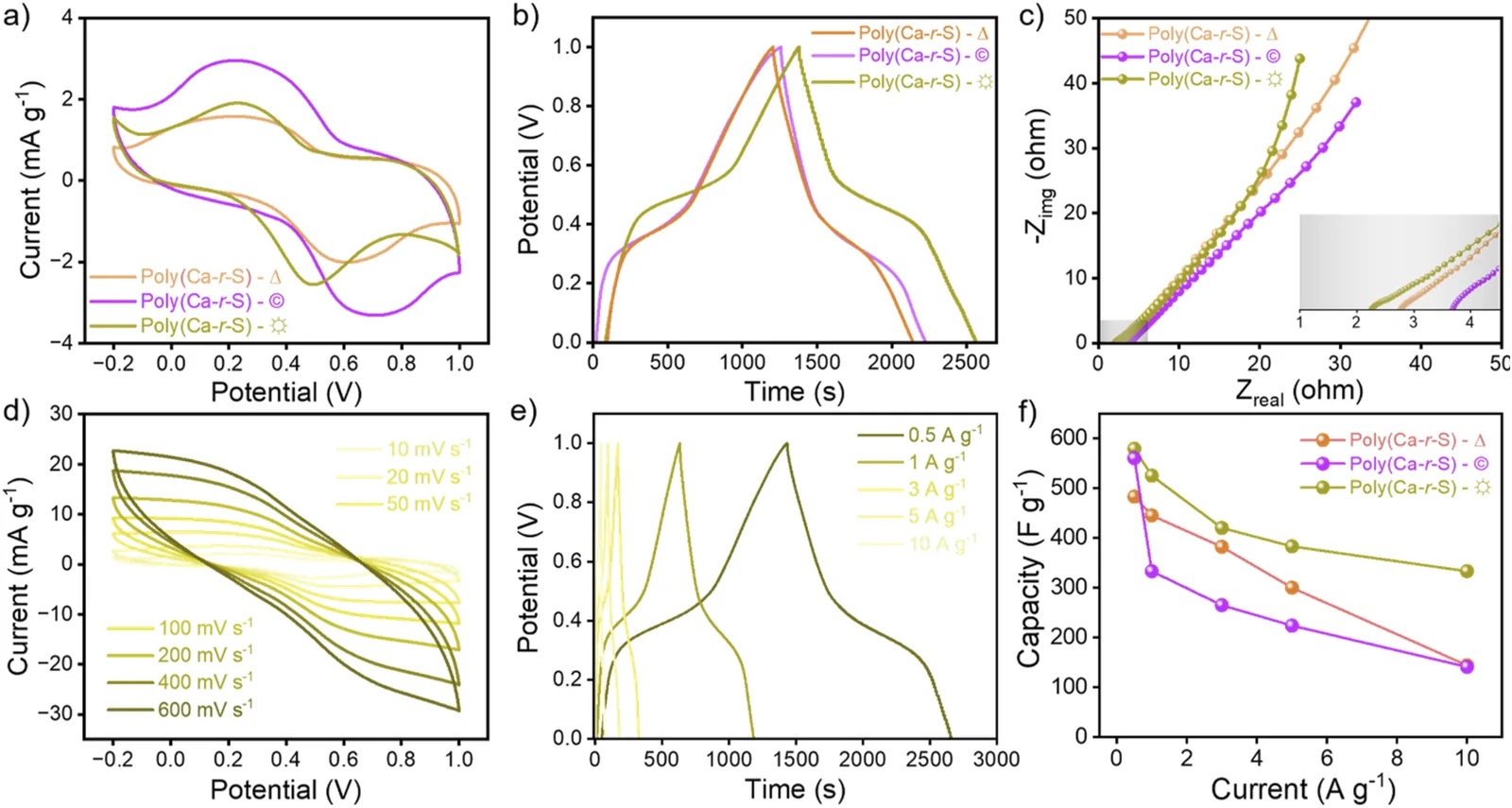
Electrochemical performance comparison of poly(Ca-*r*-S) copolymers. (a) Cyclic voltammetry (CV) curves recorded at 10 mV s^−1^. (b) Galvanostatic charge–discharge (GCD) profiles at 0.5 A g^−1^. (c) Nyquist plots obtained from electrochemical impedance spectroscopy (EIS). (d) CV curves measured at various scan rates (10–600 mV s^−1^) for poly(Ca-*r*-S) – ☼. (e) GCD curves at different current densities (0.5–10 A g^−1^) for poly(Ca-*r*-S) – ☼. (f) Capacity-retention plots correlating the energy and power density of the copolymers.

To evaluate the electrochemical performance of the synthesized copolymers, cyclic voltammetry (CV), galvanostatic charge–discharge (GCD), and electrochemical impedance spectroscopy (EIS) measurements were conducted using a three-electrode configuration in 0.5 M H_2_SO_4_ at room temperature (Fig. S24 and 6). CV curves recorded at 10 mV s^−1^ for all three copolymers (Fig. 6a) exhibited a stable potential window of 1.2 V (−0.2 to 1.0 V). The quasi-rectangular voltammograms, accompanied by well-defined redox peaks, indicate that both electric double-layer capacitance and pseudocapacitance contribute to the overall charge-storage mechanism.^54^ The appearance of redox peaks is consistent with the pseudocapacitive behavior associated with sulfur species. GCD profiles obtained at 0.5 A g^−1^ (Fig. 6b) displayed quasi-triangular shapes, further supporting the coexistence of double-layer and pseudocapacitive processes inferred from the CV results.^55^ Among the three samples, poly(Ca-*r*-S) – ☼ exhibited the longest charge–discharge duration and the highest specific capacitance (551 ± 9 F g^−1^), followed by poly(Ca-*r*-S) – © (512 8 ± 11 F g^−1^) and poly(Ca-*r*-S) – Δ (430 ± 13 F g^−1^) as determined from three independent experiments (Fig. 6b and S25). EIS analysis (Fig. 6c) revealed that poly(Ca-*r*-S) – ☼ possessed the lowest solution resistance (*R*_s_ < 2.2 Ω) and the smallest charge-transfer resistance (*R*_CT_ < 0.5 Ω), outperforming the other two copolymers. These findings demonstrate that poly(Ca-*r*-S) – ☼ facilitates more efficient charge transport and exhibits lower polarization resistance, supporting its superior overall electrochemical performance. Accordingly, detailed electrochemical studies were further carried out on the poly(Ca-*r*-S) – ☼ electrode.

Fig. 6d and e, which summarizes the CV curves recorded over a scan-rate range of 10–600 mV s^−1^, display distinct redox peaks attributed to sulfur, confirming the pseudocapacitive behavior of poly(Ca-*r*-S) – ☼. The symmetric anodic and cathodic peaks reflect highly reversible redox transitions. GCD measurements yielded specific capacitances of 579, 525, 420, 383, and 333 F g^−1^ at current densities of 0.5, 1, 3, 5, and 10 A g^−1^, respectively. The gradual decrease in capacitance at higher scan rates and current densities is attributed to limited ion mobility and incomplete utilization of active sites under fast charge–discharge conditions. Fig. 6f presents the rate capability of all copolymer electrodes, demonstrating the excellent electrochemical performance of poly(Ca-*r*-S) – ☼ compared to the other copolymers. Among them, poly(Ca-*r*-S) – ☼ delivers the highest capacitance at every tested current density and retains a larger fraction of its capacity at high rates, demonstrating better rate performance. This behavior can be attributed to the extended S–S bond chains within the polymer backbone, which create an electron-rich environment due to the lone-pair electrons on sulfur atoms. The delocalized electron density along the polysulfide chain can facilitate rapid charge transfer and provide accessible redox-active sites, thereby promoting efficient proton adsorption and diffusion during the charge–discharge process. Poly(Ca-*r*-S) – © exhibits the lowest capacitance and the steepest decline with increasing current, while poly(Ca-*r*-S) – Δ shows intermediate behavior. These differences correlate directly with the charge-transport characteristics observed in the EIS results (Fig. 6c). The lower *R*_S_ and *R*_CT_ values of poly(Ca-*r*-S) – ☼ explain its enhanced high-rate performance, whereas the higher *R*_S_ value of poly(Ca-*r*-S) – © accounts for its poorer rate capability. The current response (*i*) in the CV curve can be correlated with the scan rate using the power law relationship (*i* = *av*^*b*^) where *a* is a constant and *b* is the power-law exponent, with *b* ∼1 indicating surface-controlled redox processes and *b* ∼0.5 corresponding to diffusion-controlled behavior.^56^ CV curves at different scan rates are presented in Fig. S26a–c. The *b* value for the composite was obtained from the slope of the log(*i*) *vs.* log(*v*) curve and was found to be in the range of 0.48–0.63 over the entire potential range (Fig. S26d–f). The *b*-value indicates that the charge storage mechanism is a combination of the EDLC and faradaic redox reaction. Fig. S26g–i shows an *R*^2^ value calculated from the anodic and cathodic current close to ∼1.0, indicating the highly reversible nature of the anodic and cathodic processes.

**Fig. 7:**
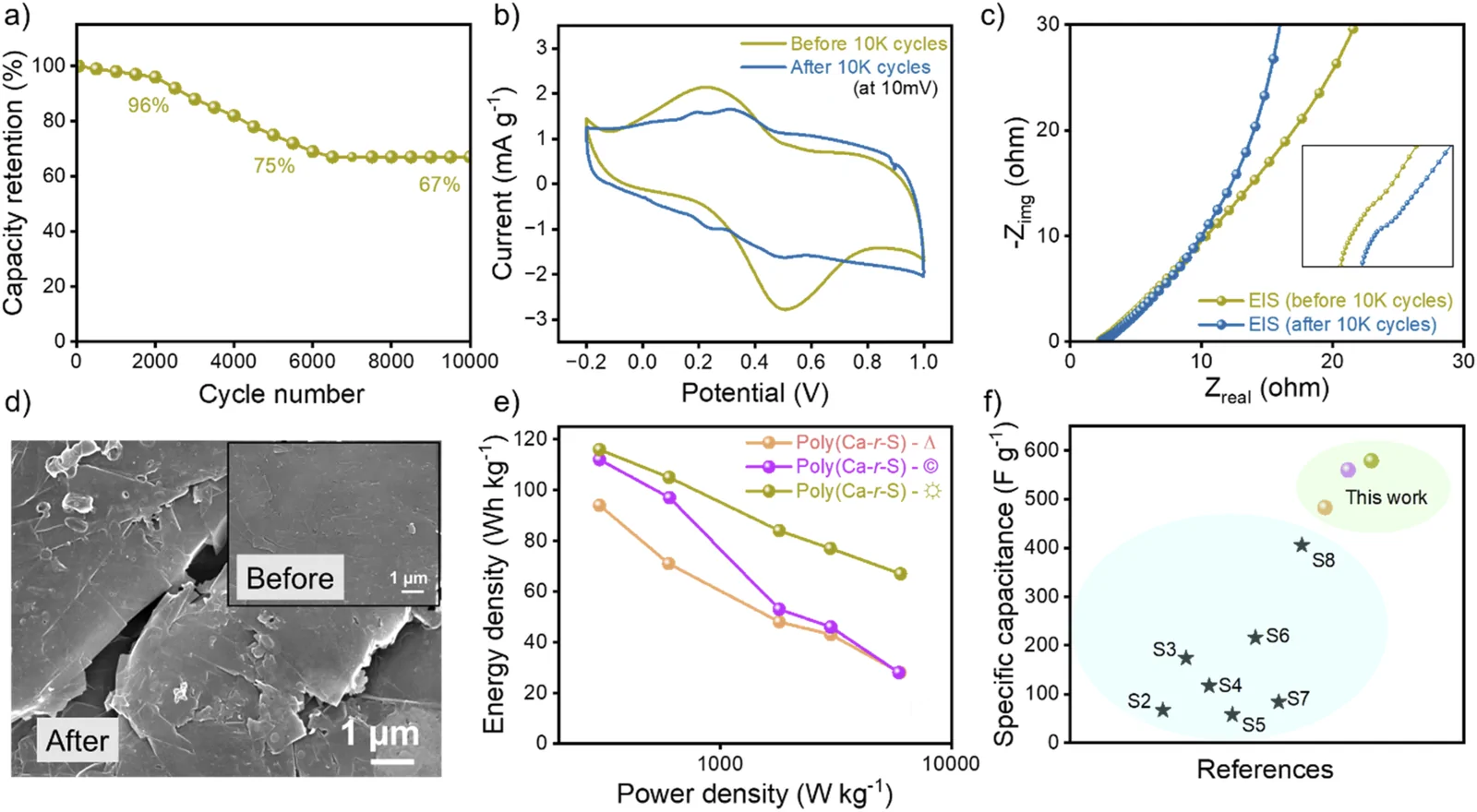
Electrochemical performance of poly(Ca-*r*-S) – ☼ as a supercapacitor electrode. (a) Cycling stability measured at a current density of 10 A g^−1^. (b) Cyclic voltammetry (CV) curves. (c) Electrochemical impedance spectroscopy (EIS) Nyquist plots recorded before and after 10 000 cycles. (d) SEM images of the electrode surface before (inset) and after 10 000 charge–discharge cycles. (e) Ragone plot comparing energy and power densities. (f) Comparative plot of specific capacitance of the reported literature on PBZ and this work.

Long-term cycling stability of the poly(Ca-*r*-S) – ☼ electrode was evaluated over 10 000 charge–discharge cycles (Fig. 7a). The electrode retained 96% of its initial capacitance after 1000 cycles, 75% after 5000 cycles, and 67% after 10 000 cycles at 10 A g^−1^. The capacity retention is 99% at 1000 cycles, and 86% after 5000 cycles at 5 A g^−1^ (Fig. S27), indicating good durability with gradual performance degradation. CV curves at 10 mV s^−1^ (Fig. 7b) before the stability test showed distinct and symmetric redox peaks, confirming pseudocapacitance. After 10 000 cycles, the redox peaks became less prominent and slightly shifted, reflecting decreased reversibility. Nyquist plots (Fig. 7c) further evidenced increased *R*_S_ and a larger semicircle after prolonged cycling, indicative of higher charge-transfer resistance and slower ion diffusion.

To elucidate the origin of the observed capacitance loss, X-ray photoelectron spectroscopy (XPS) was performed on the copolymer-coated electrodes before and after electrochemical cycling at two different current densities, (Fig. S28). As expected, exposure to the acidic electrolyte results in a pronounced XPS signal corresponding to sulfate species, indicating acid adsorption on the electrode surface. Notably, the S and C spectral regions exhibit negligible changes relative to the control electrode (immersed in sulfuric acid electrolyte), suggesting that the chemical composition and bonding environment of the copolymer remain largely preserved during cycling. In contrast, scanning electron microscopy (SEM) (Fig. 7d) reveals clear evidence of morphological degradation, with the emergence of cracks and structural discontinuities after cycling. Understanding active interfaces plays a critical role in determining the electrochemical performance and durability of energy storage devices.^57^ To elucidate the origin of the observed capacity decline upon cycling, the cross-sectional morphology of the electrode was examined. This analysis revealed a slight increase in the coating thickness after cycling, from 384 ± 4 µm to 393 ± 6 µm, compared to 337 ± 1 µm for the neat Grafoil (Fig. S29) was observed. This increase is attributed to crack propagation and localized structural rearrangements within the coating matrix during electrochemical cycling. Importantly, no evidence of coating delamination or material loss was observed. Consistent with this observation, digital photographs of the electrochemical cell before and after cycling showed no visible loss of active material (Fig. S30). Furthermore, the stability of the electrode in acidic media was evaluated by immersing it in 0.5 M H_2_SO_4_, where no discernible loss of active material was observed even after prolonged soaking. This finding is further corroborated by a control experiment, in which the copolymer-coated Grafoil exhibited no detectable residue release or leaching from the substrate even after 30 days. The combined XPS and SEM analyses indicate that the capacitance fading is primarily governed by mechanically induced degradation, specifically crack formation and interfacial stress accumulation.

Overall, the electrochemical and structural analyses demonstrate that although the poly(Ca-*r*-S) – ☼ electrode exhibits excellent initial capacitance, high-rate performance, and reasonable cycling stability, prolonged cycling leads to structural deterioration that limits ion transport and redox reversibility. The Ragone plot (Fig. 7e) further confirms that poly(Ca-*r*-S) – ☼ exhibits superior energy and power characteristics compared to the other copolymers. These findings highlight that tailoring the copolymerization pathway not only offers a sustainable synthesis approach but also significantly influences the balance between energy and power, with poly(Ca-*r*-S) – ☼ demonstrating the most promising performance for practical energy-storage applications. Fig. 7f presents a comparative analysis of the specific capacitance values reported for polybenzoxazine-based electrodes alongside the results obtained in the present study, with the corresponding data summarized in Table S9. The results clearly indicate that the pristine synthesized copolymer, poly(Ca-*r*-S), exhibits superior electrochemical performance compared with most materials previously reported. The present work demonstrates the room-temperature synthesis of a waste-derived, metal-free copolymer that exhibits significant electrochemical performance without requiring energy-intensive carbonization. Notably, the poly(Ca-*r*-S) – ☼ variant delivers exceptional specific capacitance, underscoring its potential as an efficient electrode material for high-performance supercapacitor applications.

### Fabrication and characterization of flexible copolymer-coated substrates

Flexible energy-storage devices have received considerable attention due to their applicability in a wide range of flexible and wearable electronics for medical, military, and consumer applications, including foldable smartphones, glucose and blood-monitoring devices, smart textiles, and body-mounted sensors.^58–60^ Carbon cloth has recently emerged as an attractive substrate for such devices owing to its high electrical conductivity, large surface area, mechanical robustness, thermal and chemical stability, and, importantly, its flexibility, recyclability, and compatibility with human skin. These attributes have made carbon cloth widely explored for flexible electrochemical and sensor applications.^61–63^

**Fig. 8:**
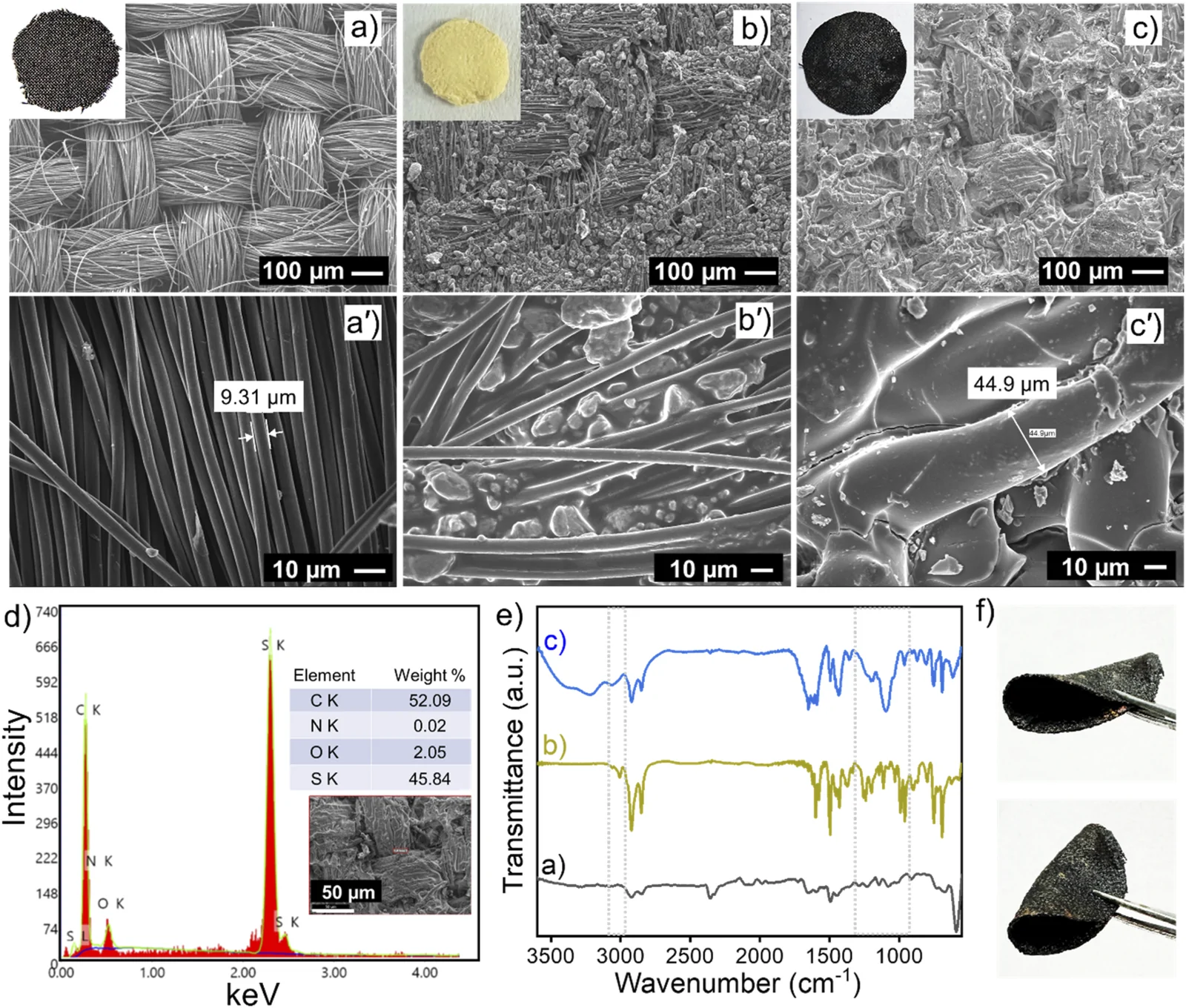
Surface and structural characterization of carbon cloth before and after copolymer coating. SEM images of (a and a′) pristine carbon cloth and (b and b′) carbon cloth after adsorption of Ca and sulfur. (c and c′) SEM images illustrating the *in situ* formation of the poly(Ca-*r*-S) – ☼ coating under light-mediated conditions at different magnifications. (d) EDX analysis of the copolymer-coated carbon cloth (200 × 200 µm^2^). (e) Stacked FTIR spectra comparing the pristine cloth, monomer-coated cloth, and the *in situ*-generated copolymer coating. (f) Digital photographs demonstrating flexibility of the copolymer-coated carbon cloth.

A light-mediated strategy was employed to achieve *in situ* coating of the poly(Ca-*r*-S) copolymer onto different substrates, including carbon cloth and filter paper (Fig. 8 and S31). SEM analyses (Fig. 8a–c and S31a–c) confirm continuous, homogeneous films across the substrates with uniform coating of the copolymer on carbon cloth and filter paper. Clearly a ∼5-fold increase in diameter of the carbon fiber is noticed, confirming that substantial loading of poly(Ca-*r*-S)@carbon fibers occurred and that too in mild-irradiation conditions, which is difficult to coat otherwise (at temperature >135 °C due to sensitivity of substrates). EDX mapping (Fig. 8d and S31d) indicated clear excellent sulfur enrichment attributable to the sulfur-based copolymer at the carbon fabric. Additionally, the cross-sectional morphology of neat *vs.* the coated carbon cloth (CC) was determined from FESEM (before and after coating), Fig. S32. Cross-sectional images of the neat CC show a three-dimensional network of interwoven carbon fibers with the average thickness of 512 ± 30 µm. Quantitative analysis of multiple cross-sectional of coated CC indicates an increase in average coating thickness to 591 ± 40 µm. Upon *in situ* copolymerization in the presence of CC, the coating adhered both to the surface and infiltrated within the CC matrix suggesting successful deposition and good adhesion to the porous substrate. To provide a visual indication of the adhesion quality, an evaluation of the extent of coating removal after making cuts through the coating layer crosshatch experiment was performed, Fig. S33. The uncoated carbon cloth exhibited removal of fine fibres or particulate debris upon tape detachment, indicating limited substrate cohesion. In contrast, the copolymer coated carbon cloth showed negligible fibre or copolymer removal, demonstrating good interfacial adhesion and integrity of the coating. FTIR (Fig. 8e and S31e) analysis further verified successful polymer incorporation on the carbon cloth/filter paper, evidenced by the disappearance of characteristic benzoxazine stretching bands and the broadening of the key vibrational features eliminating the need for an additional post-curing step. Notably, copolymer coating on the carbon cloth is more flexible and robust (Fig. 8f and S31f) than that obtained on the filter paper. Bulk composition of the coating was determined from the TGA curves (Fig. S34). A variation in the feed-in to the copolymerization ratio of Ca : S in poly(Ca-*r*-S) – ☼ was found to lie between 0.8 : 1 and 1.2 : 1, indicating heterogeneity of the coating, and largely dependent on the localized loading of the monomers.

## Conclusion

A room temperature, visible-light-mediated inverse vulcanization route has been established for the synthesis of benzoxazine–sulfur copolymer, offering a milder and more sustainable alternative to conventional thermal methods. The use of this chemical strategy enables efficient C–S bond formation without catalysts, minimizes hazardous H_2_S evolution, and improves solution processability while maintaining high sulfur incorporation. Mechanistic investigations confirm the generation of sulfur-centered radicals under irradiation and support a controlled ring-opening copolymerization pathway with reduced thiol functionality relative to thermally prepared analogues. The light-mediated copolymer demonstrates superior electrochemical performance compared to heat- and catalyst-derived materials, exhibiting high specific capacitance, favorable rate capability, and good cycling stability. Furthermore, the process enables uniform coating of flexible substrates under mild conditions, highlighting its versatility for fabricating solution-processable and flexible sulfur-rich materials. Overall, this work advances the development of sustainable sulfur-based polymer systems synthesized under energy-efficient conditions and underscores their potential for next-generation electrochemical energy-storage applications.

## Author contributions

The manuscript was written through the contributions of all authors. All authors approved the final version of the manuscript. S. Y.: methodology, investigation, writing – original draft; S. Z.: electrochemical investigation, writing – original draft; B. L.: supervision, conceptualization, funding acquisition, writing – review and editing.

## Conflicts of interest

There are no conflicts to declare.

## Supplementary Material

SC-017-D6SC04441G-s001

## Data Availability

The data that support the findings of this study are available within the article and supplementary information (SI). Supplementary information: experimental details, synthetic scheme and NMR of monomers; screening of photoinduced control reactions; control experiments; UV spectra; time-dependent ^1^H NMR spectroscopy; PXRD; HSQC, DOSY NMR spectra; Raman spectra; comparison of average molecular weights; MALDI-ToF-MS; XPS; model reaction; EPR spectra and mass spectrum of reaction mixture after irradiation; CV curves and related plot; SEM images, FTIR, and EDAX of filter paper; comparative table of polybenzoxazine reported in the supercapacitor with current work. See DOI: https://doi.org/10.1039/d6sc04441g.
