# Supplementary material for: Visible light-driven benign synthesis of benzoxazine–sulfur copolymers: high-performance materials for electrochemical applications
Source: Chem Sci. 2026 Aug 3. Online ahead of print. doi: 10.1039/d6sc04441g (PMC13430728; doi:10.1039/d6sc04441g)
Supplement: SC-OLF-D6SC04441G-s001 [file SC-OLF-D6SC04441G-s001.pdf]

Supplementary Information (SI) for Chemical Science.
This journal is © The Royal Society of Chemistry 2026

Supplementary Information

Visible Light-Driven Benign Synthesis of Benzoxazine-Sulfur Copolymers: High-Performance Materials for Electrochemical Applications

Shivani Yadav, Saad Zafar, and Bimlesh Lochab*

Materials Chemistry Laboratory, Department of Chemistry, School of Natural Sciences, Shiv Nadar University, Gautam Buddha Nagar, Uttar Pradesh 201314, India.
*bimlesh.lochab@snu.edu.in

Table of Contents

Figure S1. Digital images of reaction set-up of light-mediated synthesis of sulfur-benzoxazine copolymer.

Figure S2. a) Synthetic scheme and b) ^{1}H and ^{13}C NMR of Ca monomer.

Table S1. Screening of photoinduced control reactions.

Figure S3. Control experiments a) digital images for the corresponding color change in individual monomer post light-treatment, and b) stacked ^{1}H NMR of Ca in dark and light.

Figure S4. UV spectra of sulfur and Ca.

Table S2. Benzoxazine sulfur copolymers reported.

Table S3. Inverse vulcanization with alkene/alkyne monomers.

Figure S5. a) Time-dependent ^{1}H NMR spectroscopy of poly(Ca-*r*-S)$_6$ – ☼ was performed at 25 °C. Characteristic resonances are highlighted. (b) Percentage monomer conversion over time, represented by the decay of Ar–CH_2–N and O–CH_2–N signals. (c) Digital images of the progress of reaction with time. Recorded in $CDCl_3$ at 400 MHz with #1,3 benzoxazole as an internal standard for NMR kinetics.

Figure S6. Effect of photo-irradiation intensity (left: 100%, middle: 75%, and right: 50%) on the rROP of Ca using elemental sulfur after 3.5 h.

Figure S7. PXRD of lead sulfide formed during H_2S quantification.

Figure S8. Methylene blue assay for quantitative detection of H_2S.

Table S4. Comparative value of H_2S concentration from lead acetate test and methylene blue asaay.

Figure S9. Digital images of a) poly(Ca-*r*-S), (b-f) all synthesized copolymers (20 mg) when kept in $CHCl_3$ at different times.

Figure S10. Digital images of copolymers immersed in various solvents at different time intervals.

Figure S11. Dissolution rate of synthesized copolymers a) poly(Ca-*r*-S) - Δ, b) poly(Ca-*r*-S) - © and, c) poly(Ca-*r*-S) - ☼.

Figure S12. Stacked ^{1}H NMR of all the synthesized copolymers and Ca monomer. Photo-mediated polymerization was also carried out at for an h to compare the reaction progression with thermal mediated conditions. Structure of Ca monomer with labelled protons. Zoomed in spectra showing formation of new signals in the copolymers. Recorded in *$CDCl_3$.

Figure S13. HSQC spectrum of poly(Ca-*r*-S) - ☼.

Figure S14. DOSY NMR spectra of (a) Ca, (b) Poly(Ca-*r*-S) - Δ, (c) Poly(Ca-*r*-S) - ©, and (d) Poly(Ca-*r*-S) - ☼.

Figure S15. Stacked a) Raman spectra and b) PXRD results.

Table S5. Comparison of average molecular weights of the copolymers synthesized by different conditions as determined by GPC

Figure S16. Chromatograms of the signal intensity versus retention volume (RV) and simultaneous measurements of the a) low angle light scattering (LALS), b) right angle light scattering (RALS), and c) differential pressure viscometer (DP)

Table S6. GPC analysis of the copolymers using different detectors.

Figure S17. Stacked MALDI-ToF-MS of all the copolymers.

Figure S18. XPS survey scan of all copolymers. a-c) Wide survey, and d-f) deconvoluted C 1s spectra.

Table S7. Deconvoluted XPS sulfur spectra of the copolymers, showing the various sulfur-containing structures and their corresponding percentages.

Figure S19. (a) Storage modulus, (b) complex viscosity plots of the copolymers.

Table S8. Variation in crosslinking density (v) and tan delta of the copolymers.

Figure S20. Model reaction for thiol−disulfide exchange using DTNB.

Figure S21. (a) Control experiments performed and corresponding digital images of samples. (b) ^{1}H and ^{13}C NMR of Ca and comonomer mixture in presence of TEMPO before and after irradiation. (c) EPR spectra of TEMPO, elemental sulfur and reaction mixture post 1 h irradiation; (d) Mass spectrum of reaction mixture after irradiation.

Figure S22. Different comonomers used to probe the photochemical reaction with elemental sulfur.

Figure S23. NMR kinetics of photomediated reaction of elemental sulfur with PHfa and C_{sat}a was performed at 25 °C, and respective monomer conversion plots are presented following the the characteristic signal of Ar–CH_2–N and O–CH_2–N. Digital images of the aliquots to monitor progress of reaction with time. Stacked ^{1}H NMR spectra of sulfur reaction with DGEBA and, 1,3-DIB at two different time.

Figure S24. Electrochemical set-up for supercapacitor application.

Figure S25. Reproducibility of electrochemical performance of all synthesized poly(Ca-*r*-S) copolymers.

Figure S26. (a-c) CV curves, (d-f) determination of *b* value obtained by plotting log(*i*) vs. log(*v*), and (g-i) anodic and cathodic peaks current at various scan rates of all the synthesized copolymers.

Figure S27. Comparison of cycling stability at 5 Ag^{-1} current densities.

Figure S28. Comparison of XPS spectra. a) S 2p, b) C 1s, c) N 1s and, d) O 1s.

Figure S29. FESEM cross-section images of (a,a′) neat grafoil, and copolymer-coated grafoil electrode (b,b′) before cycling and (c,c′) after cycling.

Figure S30. Digital images of the electrochemical analysis setup: (a) before cycling, (b) after cycling, and (c) stability of the electrode in 0.5 M H_2SO_4 after 30 days.

Table S9. Metal- and carbonization-free electrochemical characterization of benzoxazine copolymers as cathode material for supercapacitor application.

Figure S31. SEM images of (a, a′) filter paper, (b, b′) filter paper adsorbed with Ca and sulfur and, (c, c′) in-situ coated poly(Ca-*r*-S) on the filter post photo-irradiation, d) FTIR of filter paper, comonomer coated filter paper and post irradiation, e) EDAX (200 × 200 μm^2) showing the homogenous coating and f) optical images of copolymer coated filter paper.

Figure S32. Cross sectional field-emission scanning electron microscope (FESEM) images of a-b) neat carbon cloth, c-d) coated carbon cloth at different magnifications.

Figure S33. Cross hatch adhesion tests. Digital images of neat carbon cloth (a–c) and coated carbon cloth (d–f) before and after incision of the substrate, and scotch tape.

Figure S34. TGA of neat carbon cloth and poly(Ca-*r*-S) coated carbon cloth.

Materials. Sulfur (S_8, sublimed powder, reagent grade, ≥99.5 %, mesh size=200) from Alfa Aesar, Cardanol (ρ =0.9272-0.9350 g cm^{-3}) was procured from Satya Cashew Chemicals Pvt. Ltd. (India), aniline and paraformaldehyde from Fisher Scientific, 2,2,6,6-tetramethylpiperidin-1-oxyl (TEMPO), 5,5-dimethyl-1-pyrroline *N*-oxide (DMPO), ferric chloride (anhydrous), *N,N*-dimethyl-1,4- phenylenediamine sulfate (98%) and deuterated

chloroform ($CDCl_3$) from Sigma Aldrich, sodium sulfate (anhydrous) and sodium hydroxide (NaOH) from Chemlabs. Zinc acetate hydrate was procured from Alfa Aesar (97%). All solvents used were AR grade and used as received. Carbon cloth (300 GSM) was purchased from Global nanotech Pvt. Ltd.

Characterization. For structure elucidation proton, carbon, distortion enhancement polarization transfer, and 2D heteronuclear single quantum correlation (^1H, ^{13}C, DEPT, HSQC, COSY), and diffusion-ordered (DOSY) nuclear magnetic resonance (NMR) spectra were obtained using a Bruker AV400 NMR, 400 MHz spectrometer. Chemical shift was referenced to Me_4Si (δ = 0.00 ppm) for ^1H NMR. The NMR acquisition and processing was done using MestReNova software. Mass spectrometry analysis was carried out using an Agilent HRMS Q-ToF 6540 Series in electrospray ionization (ESI) mode. Fourier transform infrared (FTIR) spectra were recorded on a Nicolet iS20 mid-infrared FTIR spectrometer equipped with an interferometer with a KBr/Ge-coated beam splitter and dynamic alignment and a thermoelectrically cooled (TEC) DTGS detector and an attenuated total reflectance diamond (iD5-ATR) accessory. Spectra were recorded in the range 4000−400 cm^{-1} with a resolution of 0.25 cm^{-1} and 32 scans were co-added to each spectrum. The polymerization behavior of monomers was evaluated using the differential scanning calorimeter DSC-3 (Star System, Mettler Toledo). For dynamic differential scanning calorimetry (DSC) scans, samples (4 ± 1 mg) were enclosed in hermetic aluminium pans and heated from 25 to 350 °C at 10 °C/min under a constant flow rate of nitrogen at 50 mL/min. Prior to the experiments, the instrument was calibrated for temperature and enthalpy using standard indium and zinc. Thermal equilibrium was regained within 1−2 min of sample insertion, and the exothermic reaction was considered completed when the recorder signal leveled off to the baseline. Thermogravimetric analysis (TGA) of the polymer and monomer was performed using a Mettler Toledo thermogravimetric analyzer (TGA) with a built-in gas controller (TGA2 SF/1100) and fitted

with an XP1U TGA balance (ultramicrobalance) under a 50 mL/min flow rate of nitrogen in the temperature range of 35−800 °C at a heating rate of 10 °C/min. UV−vis measurement of the copolymers dissolved in THF (stock solution: 1 mg/mL) was performed on a UV-1800 SHIMADZU spectrophotometer; spectra were recorded by using 5−10 μL of this stock solution added to water to make a final volume of 1 mL. The relative molecular weight variation of copolymers was determined using the gel permeation chromatogram (GPC) Viscotek Model 305 TDAmax fitted with a Viscotek modular differential refractive index detector (VE 3580 model). Two general mixed columns (T6000M, standard styrene-divinylbenzene copolymer, 300 × 8 mm) were maintained at 35 °C, and tetrahydrofuran (THF) was used as an eluent at a flow rate of 1 mL/min. The instrument was precalibrated with polystyrene standards, and data were analyzed using the Omnisec software. For measurement, samples were left overnight in tetrahydrofuran (8 mg/mL) and prefiltered through a PTFE filter (0.2 μm) to remove insoluble particles before injecting into the instrument. Powder X-ray diffraction (PXRD) was performed on a Bruker D8-Discover using Cu Kα radiation (λ= 0.154 nm) in the scattering range (2θ) of 10 to 80° at room temperature. The surface morphology and elemental mapping of the copolymer were examined using a field emission scanning electron microscope (FESEM), Jeol JSM-7610 Plus coupled with an energy-dispersive X-ray (EDX, AMETEK) detector, under an acceleration voltage of 15 kV. Raman spectroscopy measurements were carried out with an STR500 Airix microscope using a 532 nm laser at a power of 3 mW. The synthesized copolymers were analyzed using matrix-assisted laser desorption/ionization time-of-flight mass spectrometry (MALDI-Tof-MS). MALDI-Tof spectra were recorded on AutoflexMax, Bruker mass spectrometer with the detection of anions and cations in reflector mode with an accelerating voltage of 25 kV. The polymer solution in tetrahydrofuran (THF), prepared at a concentration of 100 mg/mL, was allowed to stand for 24 hours before proceeding with the analysis. Subsequently, sodium iodide (NaI) and α-cyano-4-hydroxycinnamic acid (CHCA) as

the matrix were added to the solution, maintaining a polymer:matrix:salt mass ratio of 5:25:1. The resulting mixture was then carefully spotted onto a ground steel MALDI target plate (MTP) from Bruker Daltonics. After allowing the solvent to evaporate by air drying the plate for 30 minutes, matrix-assisted laser desorption/ionization time-of-flight (MALDI-Tof) mass spectrometry was performed. X-ray photoelectron spectroscopy (XPS) spectra were acquired with a Thermo Fisher ESCALAB XI instrument. A monochromated Al (operating at a voltage of 15 kV with a current of 15 mA and a power of 225W) was used as the excitation source. A flood gun was used for the charge compensation purposes. Spectra were charge referenced to the C 1s adventitious carbon peak at 284.6 eV and analyzed using CasaXPS software. Photo-induced inverse vulcanization reactions of benzoxazine and elemental sulfur were performed using an OmniCure S1500 UV spot curing system (Lumen Dynamics) equipped with a high-pressure 200 W mercury vapor short arc lamp and high-power fiber liquid light guide. Specific wavelengths were selected using bandpass optical filters (320–390 nm and 400–500 nm; Edmund Optics). EPR spectra were recorded at room temperature using a JEOL JES-X320 X-band (Kyoto, Japan). Spectrometer settings were: field center 322.98 mT, microwave power 1 mW, sweep time 30 s, time constant 0.03 s, and modulation width 0.35 mT. EPR experimental data and hyperfine splitting parameters for all DMPO adducts were estimated using the ESR data processing software.

EXPERIMENTAL SECTION

Synthesis of Benzoxazine Monomer. The synthesis of C-a has been adopted from literature[1]. A mixture of cardanol (5.00 g, 16.61 mol), paraformaldehyde (1.00 g, 33. 22 mmol), aniline (1.51 mL, 16.61 mmol) was gradually heated to 80 ^{o}C and stirred for an hour, followed by heating at 90 ^{o}C for 3h. The reaction was indicated by evolution of water and color changed

from yellow to red brown. The completion of the reaction was monitored by TLC (thin layer chromatography). After the successful completion of the reaction, water (10 mL) was added, and organic layer was extracted with chloroform (25 mL). The organic layers were combined and washed with NaOH (0.5 N, 100 mL) followed by washing with water (3 x 30 mL), dried over sodium sulphate and filtered. The solvent was removed under reduced pressure to give C-a as a red oil. Yield 94%. ^{1}H NMR (400 MHz, $CDCl_3$, δ): 0.96 (CH_3,t), 1.37 [$(CH_2)_n$, m], 1.63, 2.09 ($CH_2CH=$, m), 2.57 (CH_2Ar, t), 2.88 [$CH_2(CH=)_2$, m], 4.64 (s, $ArCH_2N$–), 5.07 (–CH=CH–, dd), 5.29-5.50 (m, CH=, $CH_2=CH$–, –OCH_2N–,–$HC=CH_2$), 6.71 (ArH, s), 6.78 (ArH, d), 6.97 (ArH, m), 7.17 (ArH, d), 7.31 (ArH, m); ^{13}C NMR (125 MHz, $CDCl_3$, ppm): 50.34 ($ArCH_2N$–), 79.43 (–OCH_2N–).

Different Methods for the Preparation of Poly(Ca-*r*-S) Copolymers. Copolymers are abbreviated as poly(Ca-*r*-S) – y, where y is the condition used. For representation, symbols were used for different conditions. Δ for reaction at 180 °C, © for 135 °C with 5 wt% $Zn(DTC)_2$, and ☼ represent light mediated reaction at ambient temperature.

Thermal condition [poly(Ca-*r*-S) – Δ]: In a typical synthesis, elemental sulfur (100 mg) was placed in a 15 mL glass vial equipped with a magnetic stir bar and heated to 180 °C in a thermostatically controlled oil bath until a clear, orange, viscous melt was obtained. Cardanol-based benzoxazine (Ca, Experimental procedure and structural characterization is presented in SI, **Figure S2**) (100 mg) was then introduced directly into the molten sulfur via syringe, and the resulting mixture was stirred at 180 °C for 1 h to facilitate copolymer formation. After completion, the reaction mixture was allowed to cool gradually to room temperature, yielding poly(Ca-*r*-S) as a solid product in 93% isolated yield.

Catalyst Thermal condition (135 °C) [poly(Ca-*r*-S) – ©]: Elemental sulfur (100 mg) and zinc diethyldithiocarbamate ($Zn(DTC)_2$, 5 wt% relative to sulfur) were added to a 15 mL glass vial equipped with a magnetic stir bar. The mixture was heated to 135 °C until a homogeneous

molten phase was obtained. Subsequently, cardanol-based benzoxazine (C-a, 100 mg) was introduced into the melt, and the reaction mixture was maintained at 135 °C for 1 h with continuous stirring. Upon completion, the mixture was allowed to cool to room temperature, yielding a dark brown solid product. The copolymer was obtained in 95% isolated yield.

Light-mediated condition [poly(Ca-*r*-S) – ☼]: Equal masses of elemental sulfur (100 mg) and cardanol-based benzoxazine (Ca, 100 mg) were added to a 15 mL glass vial equipped with a magnetic stir bar. The reaction mixture was irradiated with visible light (400–500 nm) at room temperature for 3.5 h. A noticeable color change was observed after approximately 2 h, indicating progress of the photoinduced reaction. Upon completion, the mixture yielded a brown, sticky solid. The resulting polymer was used directly for characterization without further purification or curing and was obtained in 98% yield.

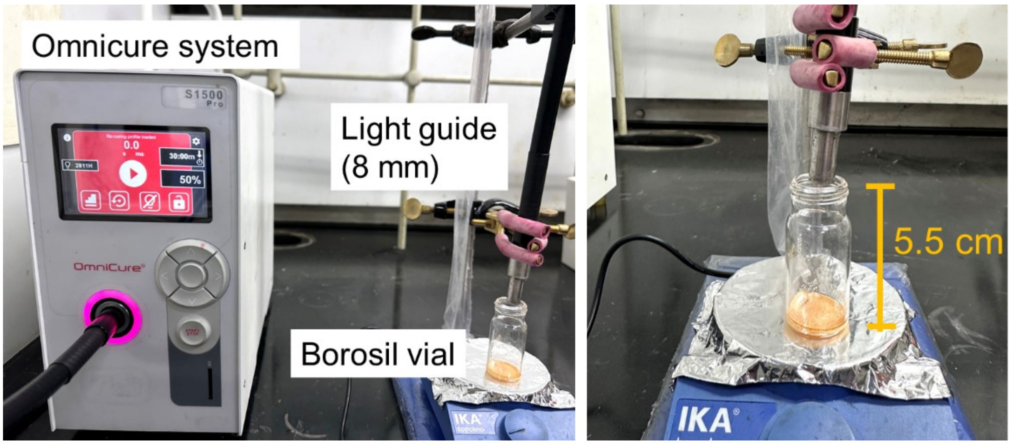

Figure S1. Digital images of reaction set-up of light-mediated synthesis of sulfur-benzoxazine copolymer. Reaction was performed in a 15 mL Borosil vial with a light guide of 8 mm diameter and variable light intensity (100%, 75% and 50%).

Time-Resolved ^{1}H NMR Kinetic Analysis of Photoinduced Sulfur–Benzoxazine Copolymerization. Equimolar amounts of Ca monomer and sulfur were placed in a 15 mL glass vial equipped with a magnetic stir bar, and the mixture was irradiated with visible light under ambient conditions. Aliquots were withdrawn at 30 min intervals and immediately diluted with $CDCl_3$ (0.6 mL). Each sample was allowed to stand for 24 h to ensure sufficient

solubility. Prior to NMR analysis, 1,3-benzoxazole (0.05 mmol) was added as an external integration standard. Monomer consumption at each time point was quantified by comparing the integrals of the benzoxazine resonances at δ 5.39 ppm (O–CH_2–N) and δ 4.64 ppm (N–CH_2–Ar) to the reference proton signal of 1,3-benzoxazole at δ 5.90 ppm, using Equation (1).

$$\% \, conversion = \frac{\int OCH_2N_{t=0} - \int OCH_2N_{t=x}}{\int OCH_2N_{t=0}} \times 100 \qquad (1)$$

where ∫OCH_2N $t = 0$ represents the integration of oxazine protons (O−CH_2−N) of the monomer at time (t = 0) and ∫OCH_2N $t = x$ represents the integration of oxazine protons of the aliquots taken at different time intervals ($t = x; x$ = 1−3.5 h).

Hydrogen Sulfide Detection and Quantification during rROP

Lead acetate test. Release of H_2S during sulfur–benzoxazine copolymerization was assessed using a lead acetate trapping setup. Elemental sulfur (S_8, 1 g) and Ca (1 g) were added to a two-neck round-bottom flask containing a magnetic stir bar. The flask was thoroughly purged with nitrogen to remove residual oxygen and moisture, after which one neck was sealed with an N_2-filled balloon to maintain an inert atmosphere. The second neck was connected to a 10 mL round-bottom flask containing an aqueous solution of lead acetate [$Pb(Ac)_2$, 5 mmol%], which served as the H_2S-trapping medium. The formation of lead sulfide (PbS) quantified by gravimetric analysis, which was correlated with the H_2S liberated.

Methylene blue assay. Methylene blue assay was carried out as described previously with some modifications[2]. A 5 mM stock solution of Na_2S in sodium phosphate buffer (20 mM, pH 7.4)/ACN (1:1) was prepared ($Na_2S.9H_2O$, 120 mg in 100 mL volumetric flask) and used as the stock solution. Aliquots of Na_2S stock solution (20, 40, 60, 80, 100, 120, 140, 160, 180, 200, 400 and, 600 μL) were added into a 10 mL volumetric flask and dissolved in a mixture of sodium phosphate buffer/ACN to obtain the respective standard solutions (10, 20, 30, 40, 50,

60, 70, 80, 90, 100, 200, 300 μM respectively). A 0.5 mL aliquot of the respective solution was reacted with the methylene blue (MB^+) cocktail: 30 mM $FeCl_3$ (200 μL) in 1.2 mM HCl, 20 mM of *N,N*-dimethyl-1,4- phenylenediamine sulfate (200 μL) in 11.4 mM HCl, 1% w/v of $Zn(OAc)_2$ (100 μL) in H_2O at room temperature for 5 min. The absorbance of methylene blue was measured at λ_{max} = 665 nm. To obtain the molar absorptivity of (MB^+) a linear regression was plotted with the observed absorbance and concentration.

Quantification of H_2S release during sulfur–benzoxazine copolymerization reaction was performed separately under three different specified reaction conditions (heat/ heat + catalyst/ light) under nitrogen atmosphere. Briefly, sulfur (S_8, 1 g) and Ca (1 g) were added to a two-neck round-bottom flask containing a magnetic stir bar. The liberated H_2S was collected in another round bottom flask containing an equivolume solution of buffer/ACN and methylene blue cocktail (2 mL, 1:1 v/v). Post completion of the reaction, absorbance was recorded using a UV-vis spectrophotometer. Following the reported protocol, the H_2S concentration was calculated using the standard Na_2S calibration curve.

Solubility Assessment of Sulfur–Benzoxazine Copolymers. For each sample, 10 mg of the copolymer [poly(Ca-*r*-S) – Δ or poly(Ca-*r*-S) – © or poly(Ca-*r*-S) – ☼] was placed in a glass vial, followed by the addition of 1 mL of solvent (high-polarity to low-polarity: DMSO, DMF, ACN, MeOH, EtOH, acetone, DCM, THF, EtOAc, diethyl ether, toluene, hexane). The vials were sealed and left undisturbed for 24 h at room temperature and solubility behavior is commented as complete dissolution, partial swelling, or insoluble for each copolymer–solvent.

Dissolution rate. A 20 mg copolymer was separately immersed in 5 different solvents (ACN, MeOH, DCM, toluene, hexane), and after every 10 min residue was filtered, dried, and weighed (m). The dissolution rate (D_s) was calculated using Equation (2).

$$D_s = \frac{20 - m}{20} \times 100\% \quad (2)$$

Rheological measurement. An MCR302e (Anton Paar) rheometer fitted with parallel plate geometry and a convection temperature device (CTD−600) was used to perform the studies. Copolymer was placed on lower measuring plate (aluminium, diameter = 25 mm) and sandwiched with the upper plate (aluminium, diameter = 8 mm) with a separation distance of 0.5 mm. The sample was cooled at a rate of 2 °C min^{-1} from 25 to -30 °C for all the copolymers, and kept for 15 min. Further the sample was heated from -30 to 100°C with amplitude $\gamma = 0.5\%$ and a frequency of 1 Hz to obtain the storage (G') and loss (G'') modulus. The cross-linking density ($\boldsymbol{v}$) for each copolymer was calculated using Flory's Equation (3).

$$\boldsymbol{v} = \boldsymbol{G'/RT} \quad (3)$$

where G' is the storage modulus chosen at the rubbery plateau, R is the gas constant, and T is the temperature (273 K).

Thiol Quantification by Ellman's Reagent (DTNB) Assay. Thiol content in the copolymers was quantified using Ellman's reagent (DTNB) following a standardized UV–Vis-based colorimetric protocol.[3] A 1000 µM stock solution of DTNB was prepared in DMSO. For each measurement, a 100 µM solution of the copolymer was prepared and mixed with an aliquot of the DTNB solution. The reaction mixture was stirred at 25 °C for 1 h to allow complete formation of the TNB^{2-} chromophore. After incubation, UV–Vis spectra were recorded, and thiol concentration was calculated using a calibration curve generated from known concentrations of 4-methoxythiophenol. Standard solutions of DTNB and 4-methoxythiophenol (various concentrations) were prepared, incubated for 1 h under identical conditions, and analyzed by UV–Vis spectroscopy. The absorbance of the TNB^{2-} peak was

converted to thiol concentration using the calibration curve. All measurements were performed in triplicate to ensure reproducibility.

In Situ Formation and Coating of Benzoxazine–Sulfur Copolymer. Cardanol-based benzoxazine monomer (Ca, 50 mg) and elemental sulfur (50 mg) was uniformly mixed to form a paste which was applied onto the surface of carbon cloth/filter paper (2 cm diameter). The sulfur-loaded substrate was placed in a petri-dish and irradiated with visible light (450–500 nm) on both sides at room temperature for 3.5 h. Irradiation induced the formation of a uniform dark brown copolymer film on the substrate. The resulting substrate was used directly for characterization without additional treatment.

EPR Spin Trapping Experiment. Separately, powdered elemental sulfur and a 1:1 w/w mixture of Ca and S_8 were charged in glass vials (5 mL) containing stir bars. To each vial, DMPO (0.2 mL, 20 mM in toluene) was added. The mixture was stirred for a min and then transferred to quartz EPR capillary tubes. Contents were irradiated with light of wavelength 400–500 nm at 25 °C for 20 min prior recording EPR. Respective control experiments, trap-free conditions and light dependence, were also conducted. Likewise, experiments were performed using TEMPO in bulk conditions.

Electrochemical measurements. All the electrochemical analyses of the electrode materials were tested on a CHI, VMP3e electrochemical workstation through cyclic voltammetry (CV), galvanostatic charge– discharge (GCD), and electrochemical impedance spectroscopy (EIS) measurements. A three-electrode system was used to investigate the electrochemical properties of individual electrodes, which were used as a working electrode, with a platinum wire as the counter electrode and Ag/AgCl as a reference electrode. The three-electrode testing was performed using 0.5 M H_2SO_4 solution. The working electrode was prepared by drop casting polymer solution on grafoil and dried at 60 °C for 12 h under vacuum. The specific capacitance

(C, F g^{-1}) of the electrode was measured from GCD (C_p) curves and calculated according to Equation (4):

$$C_P = \frac{I\,.\,\Delta t}{m\,.\,\Delta V} \tag{4}$$

where I is the discharge current (A), Δt is the discharge time (s), ΔV is the potential window (V), and m is the active mass per electrode (g). The energy density, E_d (W h kg^{-1}), and power density, P_d (W kg^{-1}), were calculated using Equation (5) and (6), respectively:

$$E_d = \frac{C_P \times \Delta V^2}{7.2} \tag{5}$$

$$P_d = \frac{E_d \times 3600}{\Delta t} \tag{6}$$

Crosshatch experiment. Adhesion of the coating was evaluated using a crosshatch adhesion test performed, **Figure S33**, with an MGW Precision Cross Hatch Cutter Adhesion Tester in accordance with ASTM D3359.[4] On a copolymer coated substrate (diameter = 2 cm), cuts of 1 mm were made in one steady motion using the cutting tool to make the cutting edge reaching the substrate with sufficient pressure. After making the cuts, surface of the substrate was brushed off lightly. The scotch tape was placed over the cuts made and rubbed firmly. The tape was removed and digital images were recorded of the tape and the substrate (before and after test) to provide a visual comparison.

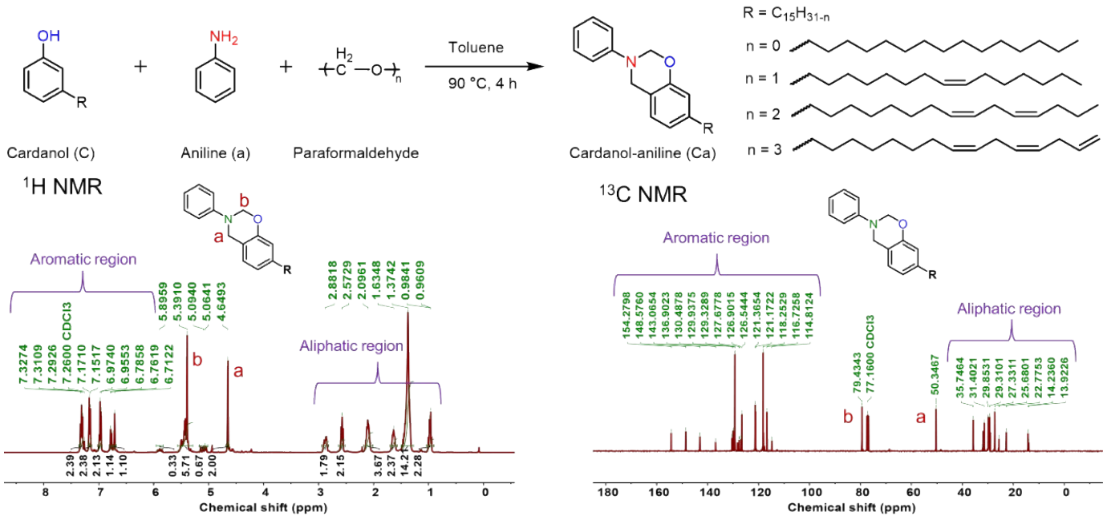

Figure S2. a) Synthetic scheme and b) ^{1}H and ^{13}C NMR of Ca monomer.

Table S1. Screening of photoinduced control reactions.

Entry	Reactants	Wavelength of light (nm)*	Observations
1	Ca + S_8*	Dark	No colour change, no reaction
2	Ca + S_8*	320-390	Unreacted sulfur, incomplete conversion
3	Ca + S_8*	400-500	Dark brown colouration, complete conversion
4	Ca	400-500	No change
5	S_8	400-500	Colour change from light yellow to turmeric solid

* 1:1 w/w; † Each reaction performed for 3.5 h

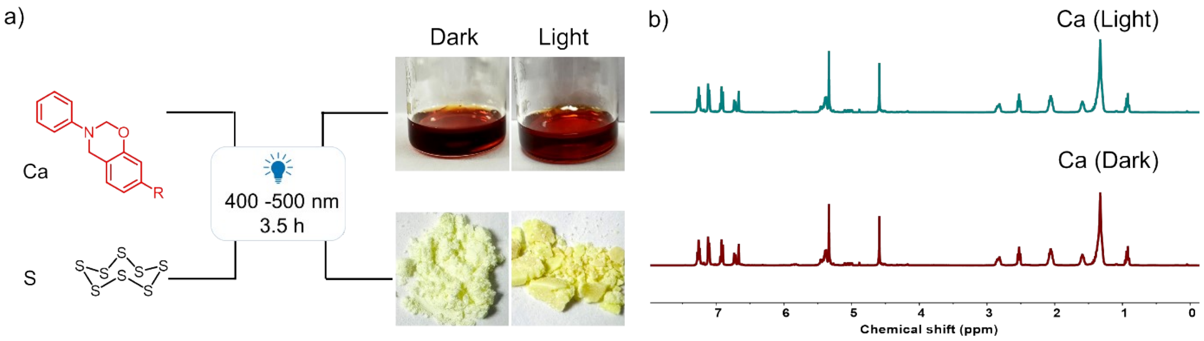

Figure S3. Control experiments a) showing digital images for the corresponding color change in individual monomer post light-treatment, and b) stacked ^{1}H NMR of Ca in dark and light.

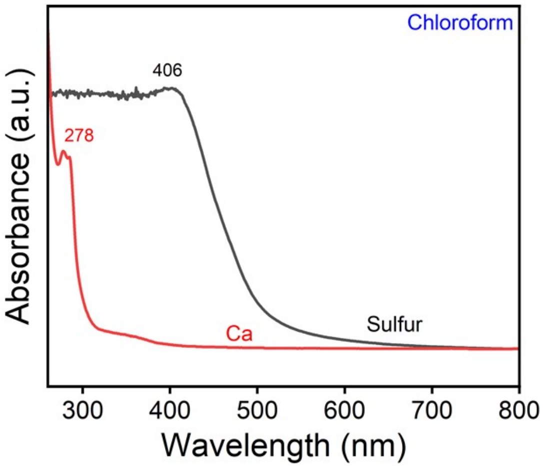

Figure S4. UV spectra of sulfur and Ca.

Table S2. Inverse vulcanized benzoxazine sulfur copolymers reported.

Copolymer	***Benzoxazine monomer***	***Temperature (°C)***	***Time***	***H_2S***	***Solubility***	***GPC***
			Synthesis			
Poly(BA-ala-*co*-S)[5]	Allyl-functional benzoxazine (BA-ala)	180	30 min	NR	Soluble at low S feed; insoluble network at ≥20 wt% S	Reported for soluble fractions
Poly(P-a-co-S)[6]	sulfur, PBz and 10	170	Up	Qualitatively	Reported only for	Reported

	mL diglyme		to 30 min	determined by lead acetate paper	soluble fractions in diglyme, ltd solubility	for soluble fractions (Mn 4092, Mw 6311)
Poly(BzPPO BA-ala-co-S)[7]	Allyl benzoxazine (BA-ala)	185	~30 min	NR	Insoluble cross-linked films	NR
Poly(Bz-r-S) (R^2S^2)[8]	Cardanol–cystamine benzoxazine	180	10 min	NR	Reprocessable covalent adaptable network	NR
Poly(Bz/S/ ionone)[9]	Renewable benzoxazine + ionones	185	30 min	NR	Insoluble thermoset*	NR
Cardanol-BZ–S copolymer[10]	Cardanol-based benzoxazine	185	~10–30 min	NR	Insoluble thermoset*	NR
Poly(S-*r*-IE-fa)[11]	Room-temperature sulfur knitting (using sodium polysulfide - ionic mechanism)	~25 (RT) and 50 °C	1 h	Qualitatively determined by lead acetate paper	Solution-processable	Reported

**depend on sulfur loading, NR: Not reported*

Table S3. Inverse vulcanization with alkene/alkyne monomers.

Synthetic approach	Alkene/alkyne comonomers	Temperature (°C)	Time	H_2S	Solubility	GPC
Thermal[12]	DIB, DVB, Styrene	≥159	High energy; solvent-free; requires crosslinker	No quantification and qualitative determination	Ltd. solubility	Only soluble fractions

			s with high boiling points, which are thermally stable			
Heat + catalysts (Metal dithiocarbamates (Zn, Fe, Co, Ni)[13]	Acrylates (e.g., ethylene glycol dimethacrylate)	135	Reduces pr oduction; enables reaction with unreactive crosslinker s.	Quantified using H2S detector	Majorly Insoluble	Only soluble fraction s
Mechanochemical [14]	Limonene, isoprene, DMBT	Room temp	3 h	Quantified	Insoluble copolyme r	No GPC
Light-mediated[15]	Low boiling point alkenes and alkynes	Room temp	6 h	Quantificatio n using Lead acetate test	Partial soluble	Only soluble fraction

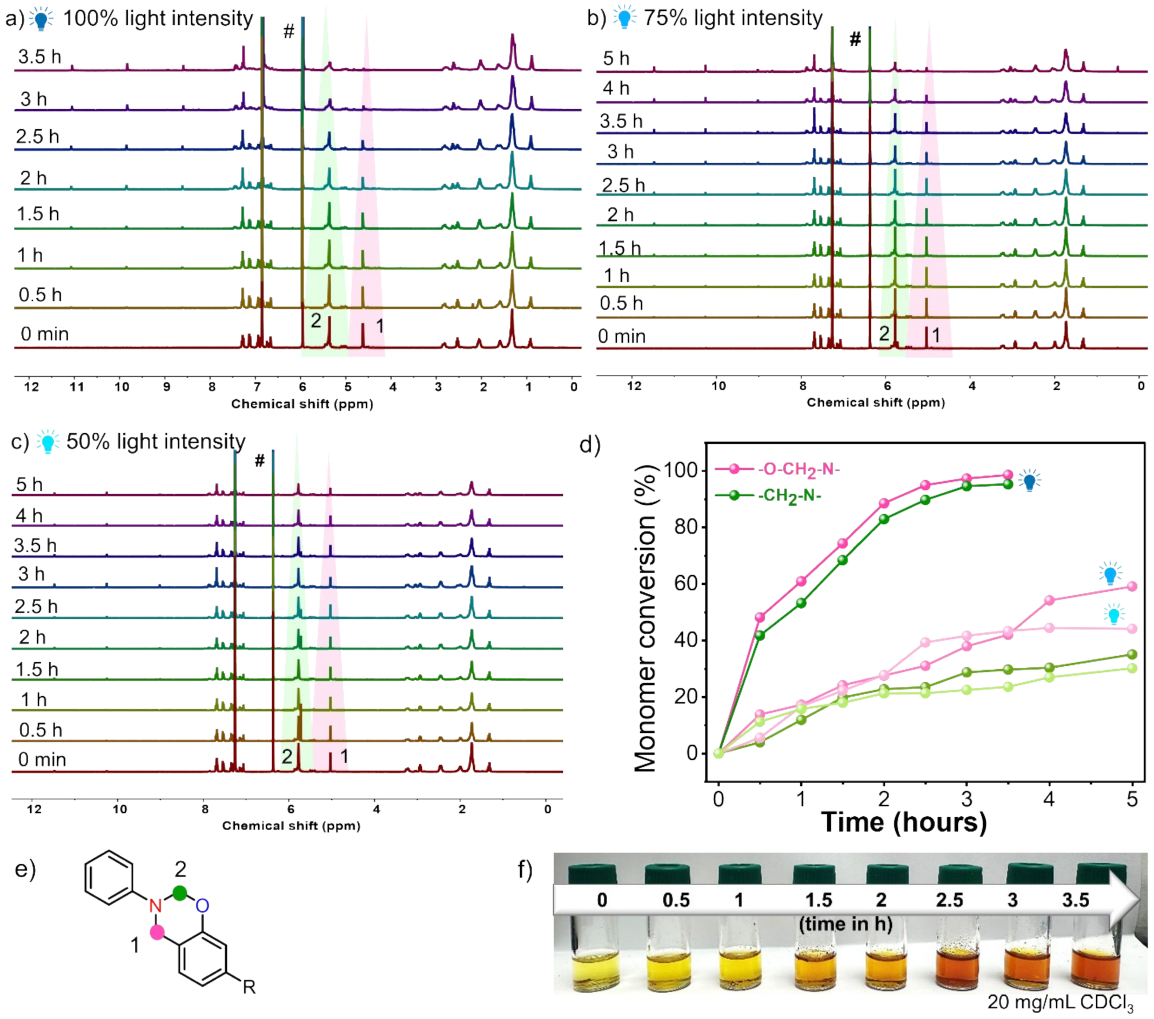

Figure S5. a) Time-dependent ^{1}H NMR stacked spectra of co-reaction of Ca and S performed at 25 ^{o}C with different light intensity a) 100% (14.5 W/cm^2), b) 75% (10.9 W/cm^2), and c) 50% (7.3 W/cm^2). Characteristic oxazine-ring resonances are highlighted. (d) Percentage monomer conversion over time at different light intensity, calculated from the decay of Ar–CH_2–N and O–CH_2–N signals. Recorded in $CDCl_3$ at 400 MHz with $^{\#}$1,3 benzoxazole as an internal standard. e) Ca marked with characteristic oxazine-ring reaction centres, f) Digital images of the progress of poly(Ca-*r*-S) – ☼ reaction with time.

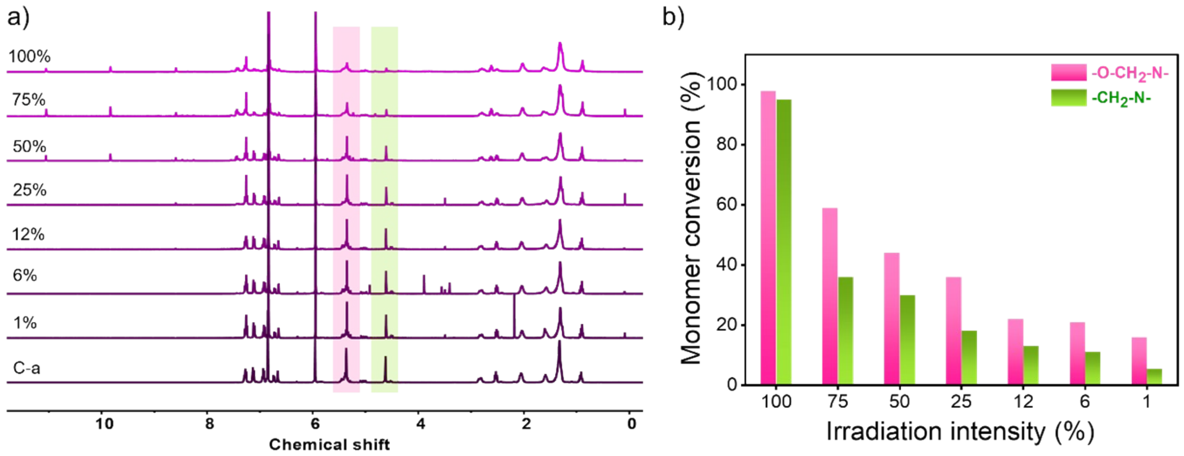

Figure S6. Effect of photo-irradiation intensity on the rROP of Ca using elemental sulfur after 3.5 h.

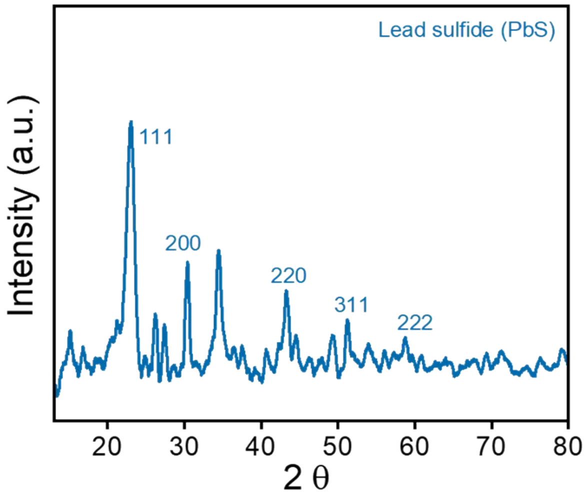

Figure S7. PXRD of lead sulfide formed during H_2S quantification. JCPDS card No. 05-0592.

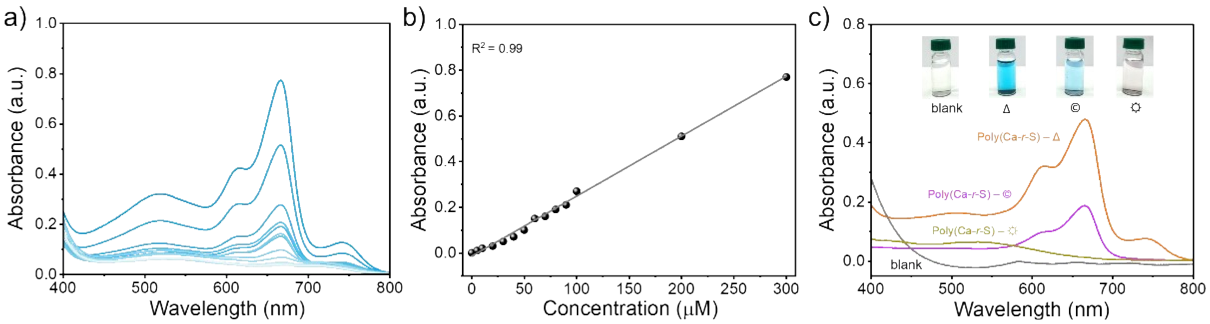

Figure S8. Methylene blue assay for quantitative detection of H_2S. (a,b) UV–Vis absorption spectra and corresponding calibration curve obtained at varying Na_2S concentrations. (c) UV–Vis spectra of the methylene blue assay for the synthesized copolymers. (inset shows digital images of the methylene blue assay corresponding to H_2S evolution under three different conditions).

Table S4. Quantitative H_2S estimation under different reaction conditions of copolymerization.

Conditions	Methylene blue assay
	H_2S (ppm)
Heat	25.9
Heat + catalyst	10.2
Light	1.0

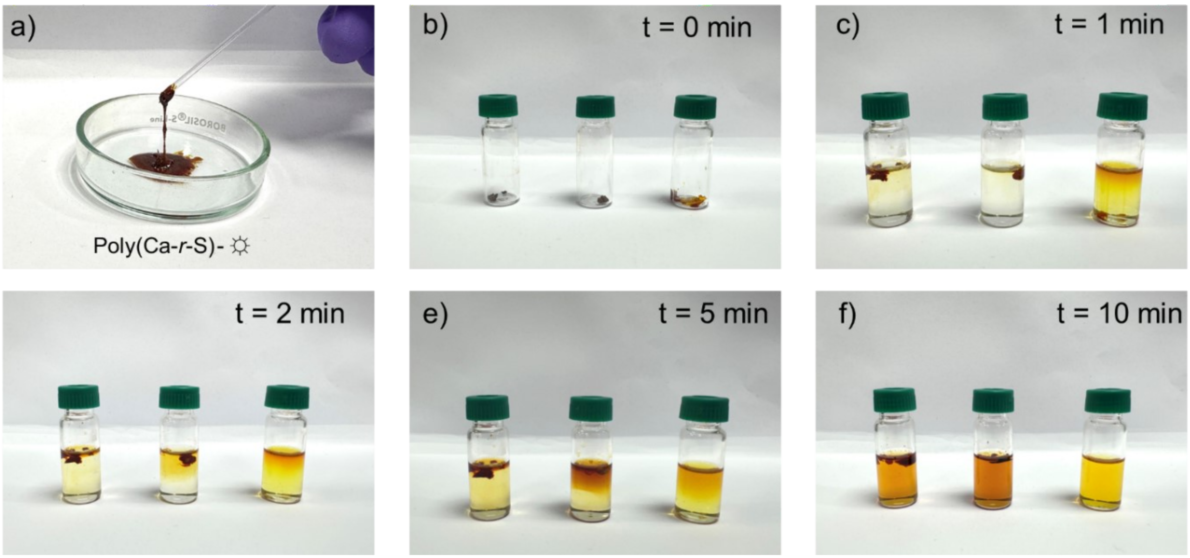

Figure S9. Digital images of a) poly(Ca-*r*-S), (b-f) all synthesized copolymers (20 mg) when kept in $CHCl_3$ at different time. Polymer concentration (20 mg mL^{-1}).

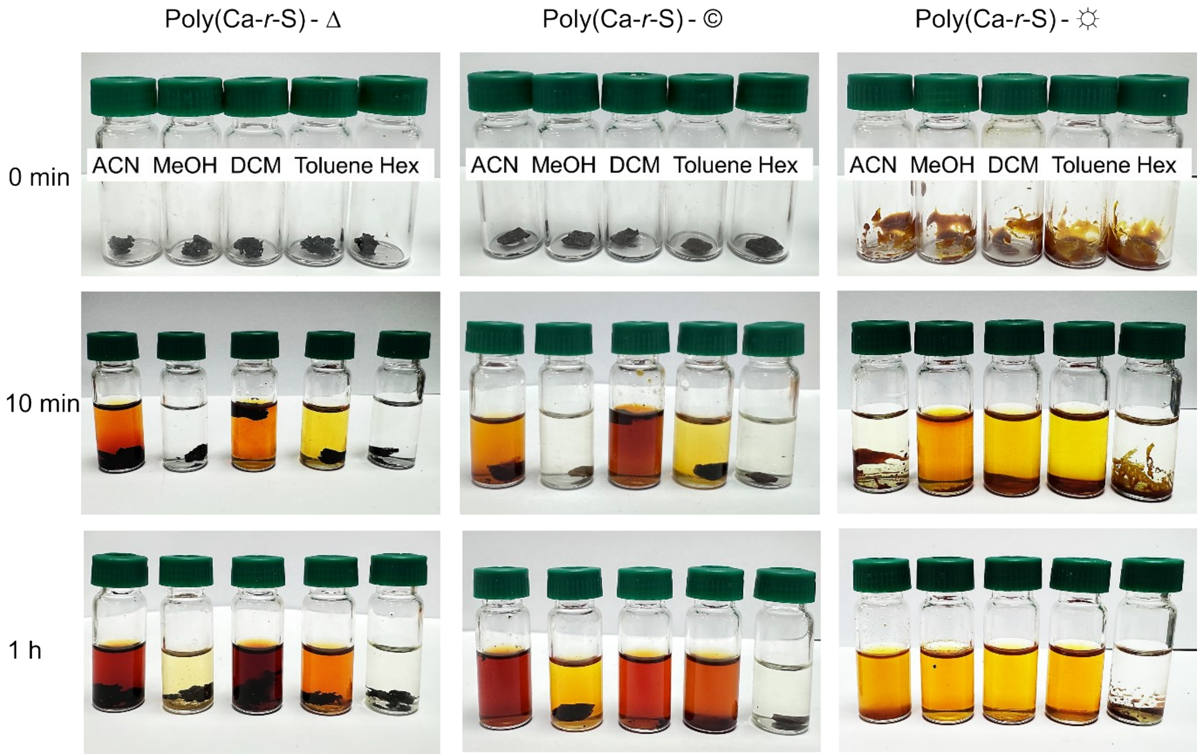

Figure S10. Digital images of copolymers immersed in various solvents at different time intervals.

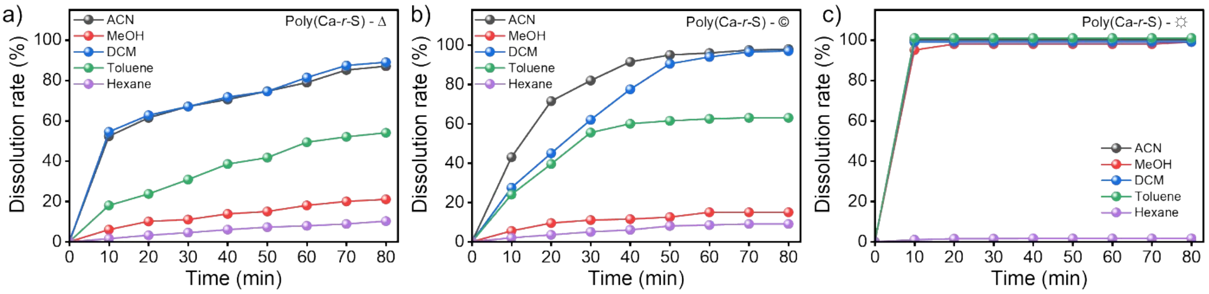

Figure S11. Dissolution rate of synthesized copolymers a) poly(Ca-*r*-S) - Δ, b) poly(Ca-*r*-S) - © and, c) poly(Ca-*r*-S) - ☼.

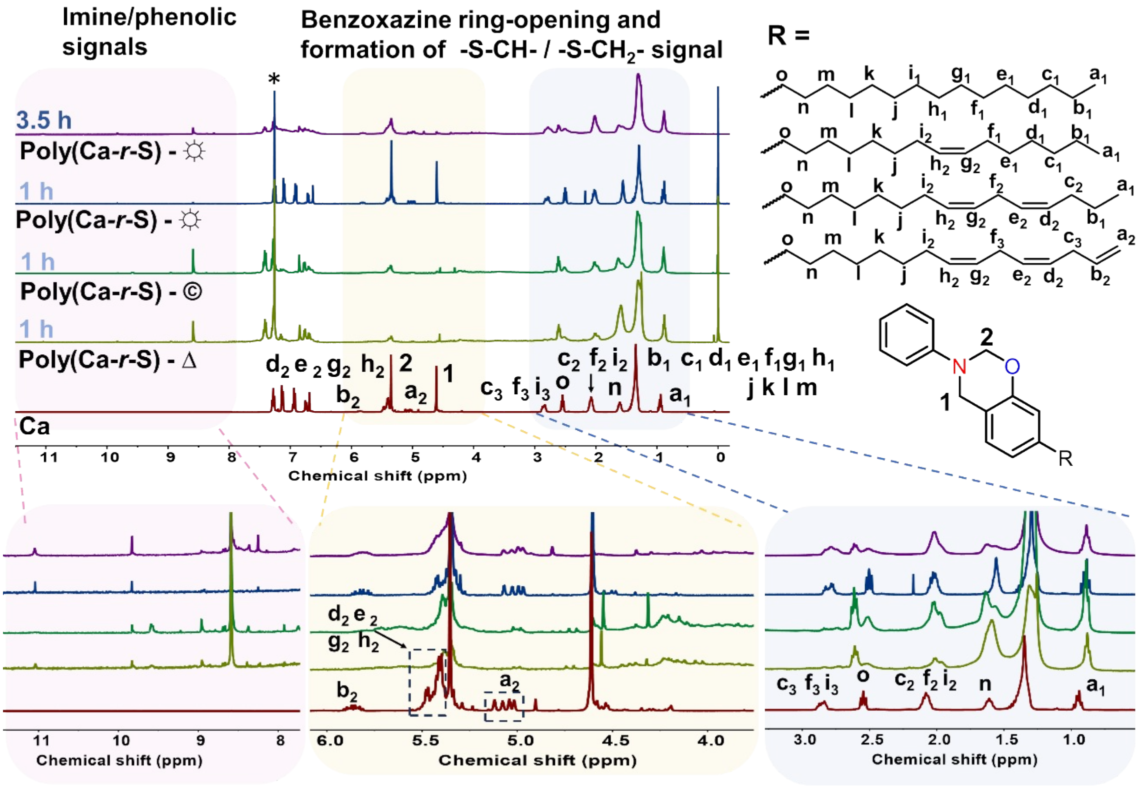

Figure S12. Stacked ^{1}H NMR of all the synthesized copolymers and Ca monomer. Photo-mediated polymerization was also carried out at for an h to compare the reaction progression with thermal mediated conditions. Structure of Ca monomer with labelled protons. Zoomed in spectra showing formation of new signals in the copolymers. Recorded in *$CDCl_3$.

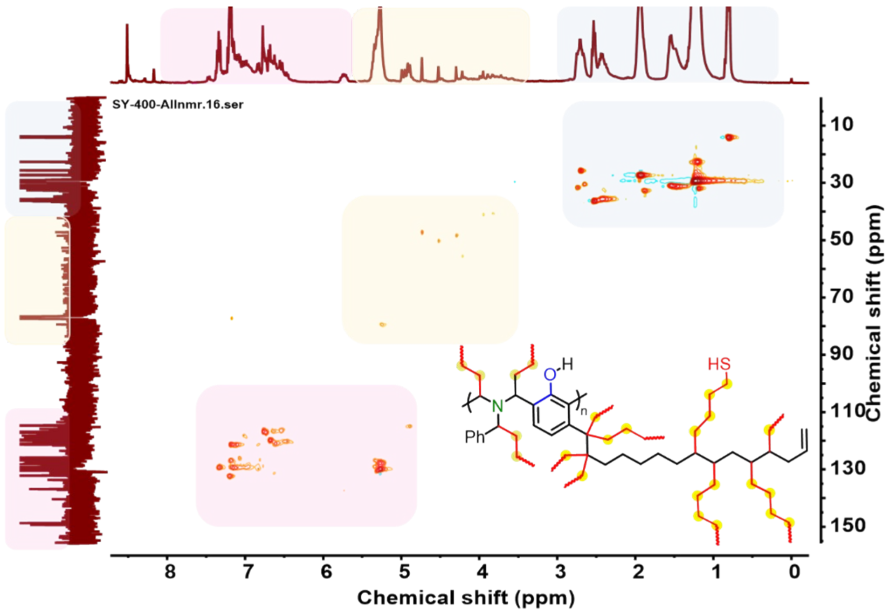

Figure S13. HSQC spectrum of poly(Ca-*r*-S) - ☼.

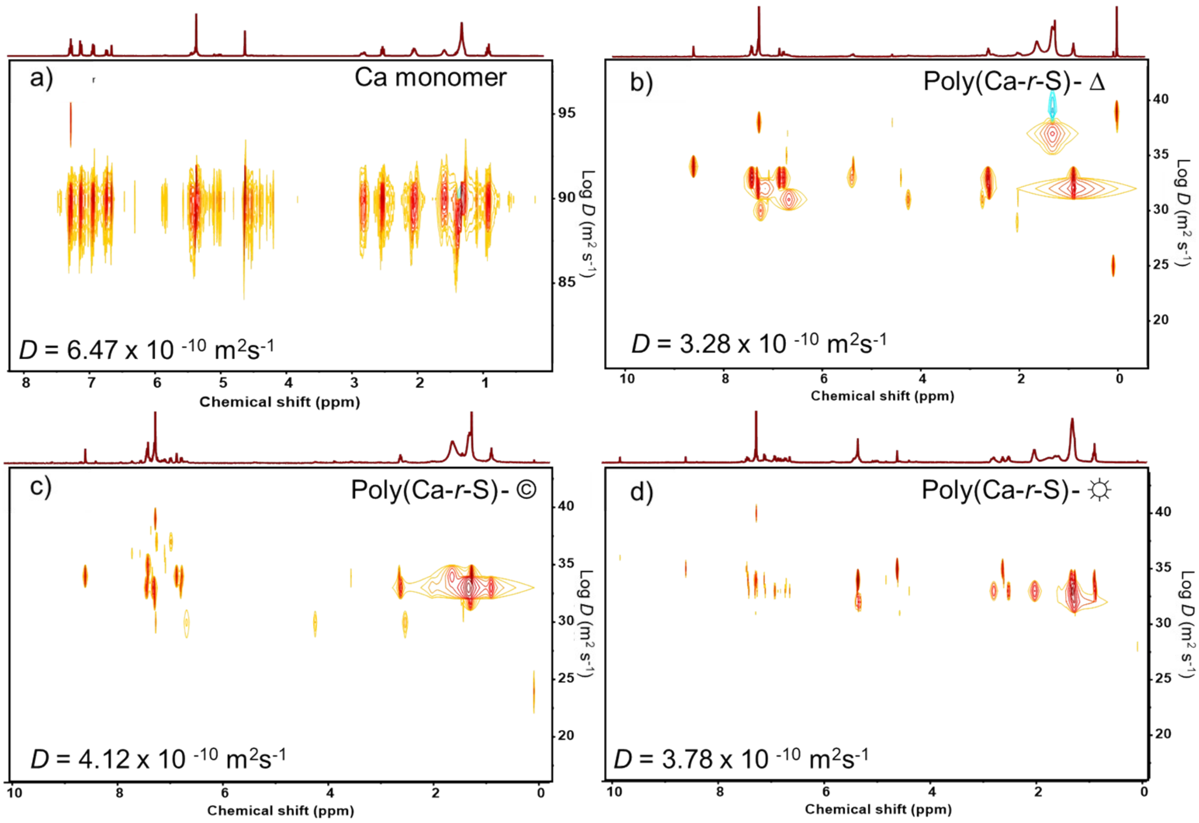

Figure S14. DOSY NMR spectra of (a) Ca, (b) Poly(Ca-*r*-S) - Δ, (c) Poly(Ca-*r*-S) - ©, and (d) Poly(Ca-*r*-S) - ☼.

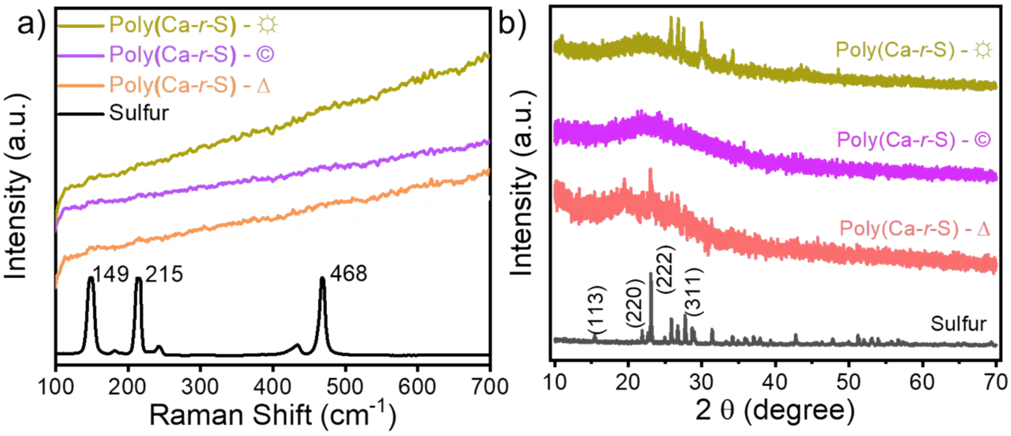

Figure S15. Stacked a) Raman spectra and b) PXRD results.

Table S5. Comparison of average molecular weights of the copolymers synthesized by different conditions as determined by GPC.

Molecular weight (g mol $^{-1}$)	Ca	Poly(Ca-*r*-S) - Δ	Poly(Ca-*r*-S) - ©	Poly(Ca-*r*-S) - ☼
$\bar{M}_n$	777	21,916	10,873	15,514
$\bar{M}_w$	876	29,265	26,614	25,462
$\bar{M}_z$	1168	36,738	81,789	49,527
$Đ$	1.1	1.3	2.4	1.6

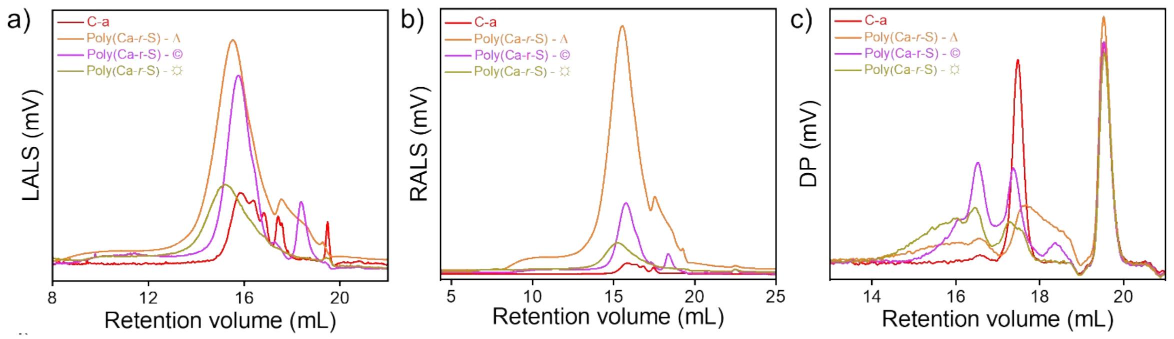

Figure S16. Chromatograms of the signal intensity versus retention volume (RV) and simultaneous measurements of the a) low angle light scattering (LALS), b) right angle light scattering (RALS), and c) differential pressure viscometer (DP).

Table S6. GPC analysis of the synthesized copolymers using different detectors.

Type of Detector	GPC data	Ca	Poly(Ca-*r*-S) - Δ	Poly(Ca-*r*-S) - ©	Poly(Ca-*r*-S) - ☼	Inference
Refractive Index	$\bar{M}_n$(g mol^{-1})	777	21,916	10,873	15,514	Relative molecular weight to PS standards. Heat has highest MW
	$\bar{M}_w$(g mol^{-1})	876	29,265	26,614	25,462	
	$\bar{M}_Z$(g mol^{-1})	1168	36,738	81,789	49,527	
	Đ	1.1	1.3	2.4	1.6	
Viscometer	M*p* (g/mol)	688	11918	6,655	7,942	Value determined is highest for heat-mediated copolymer.
	M-H a	-	0.055	0.063	-0.067	Value is very low to comment
	M-H logK (dL/g)	-	-1.027	-1.793	-1.212	Measure magnitude of the viscosity contribution. In general, sulfur copolymers are relatively compact.
	IV*n* (dL/g)	0.0258	0.1497	0.0297	0.0332	Viscosity is highest in heat, supporting higher molecular weight and branched structure.
Light scattering	Rh(η)n (nm)	0.69	3.96	1.81	2.09	Absolute molecular weight is highest in heat followed by light and then catalyst. Heat mediated copolymers have high mol wt. and higher degree of branching.
	Rh(η)w (nm)	0.7	4.11	2.06	2.21	
	Rh(η)z (nm)	0.75	4.26	2.48	2.35	

Relative molecular weights w.r.t. PS standards; IVn: Intrinsic viscosity; Rh: hydrodynamic radii

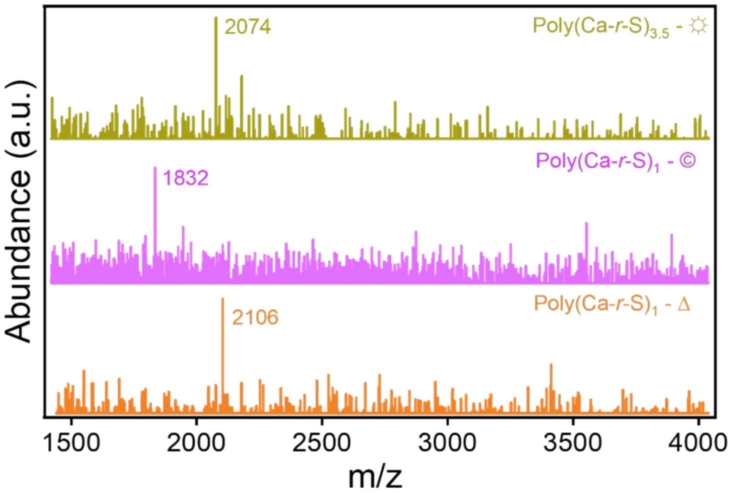

Figure S17. Stacked MALDI-ToF-MS of all the copolymers.

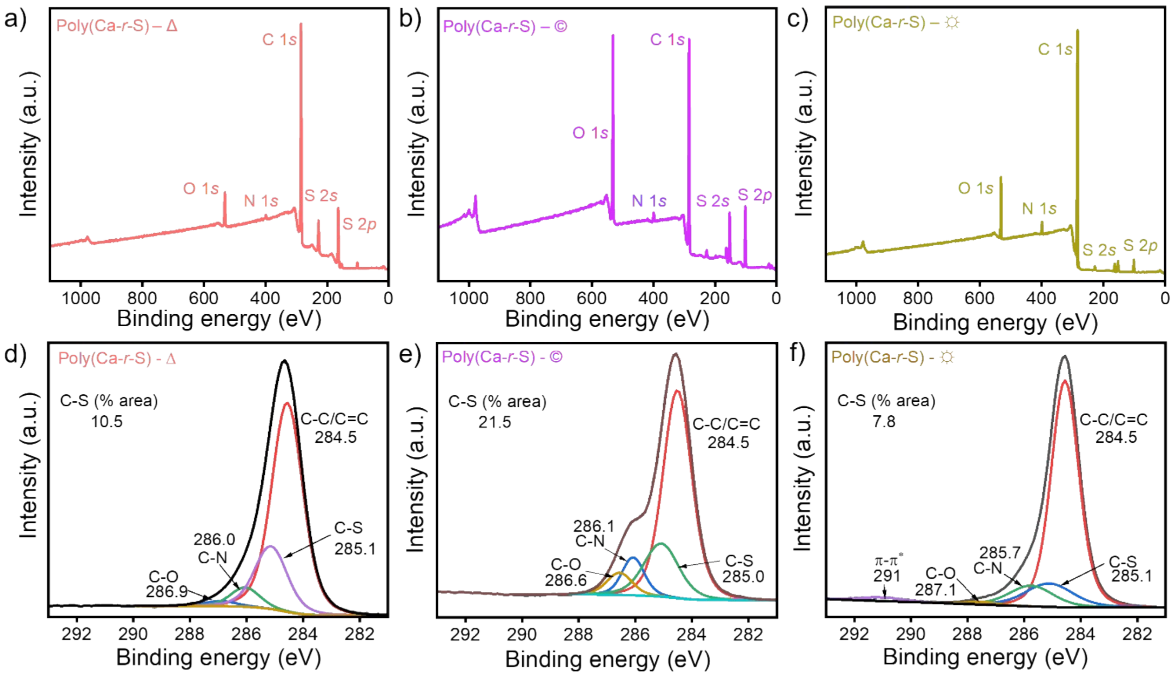

Figure S18. XPS survey scan of all copolymers. a-c) Wide survey, and d-f) deconvoluted C 1s spectra.

Table S7. Deconvoluted XPS sulfur spectra of the copolymers, showing the various sulfur-containing structures and their corresponding percentages.

Copolymer	S-S		S-H		S-C		SOx
	$2p_{3/2}$ (%)	$2p_{1/2}$ (%)	$2p_{3/2}$ (%)	$2p_{1/2}$ (%)	$2p_{3/2}$ (%)	$2p_{1/2}$ (%)	(%)
Poly(Ca-*r*-S) - Δ	47.3	23.7	11.8	5.9	7.0	3.5	0.8
Poly(Ca-*r*-S) - ©	38.0	19.0	5.6	2.8	14.3	7.2	13.1
Poly(Ca-*r*-S) - ☼	52.7	26.4	3.9	2.0	5.2	2.6	7.3

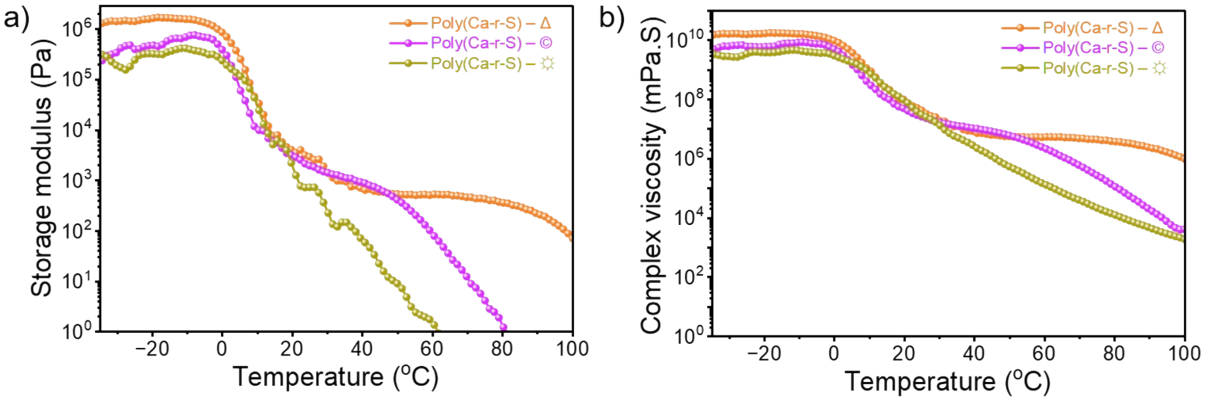

Figure S19. (a) Storage modulus, and (b) complex viscosity plots of the copolymers.

Table S8. Variation in crosslinking density of the copolymers.

**Copolymer**	**Crosslink density (mol/m^3)**
Poly(Ca-*r*-S) - Δ	375.76
Poly(Ca-*r*-S) - ©	170.38
Poly(Ca-*r*-S) - ☼	103.39

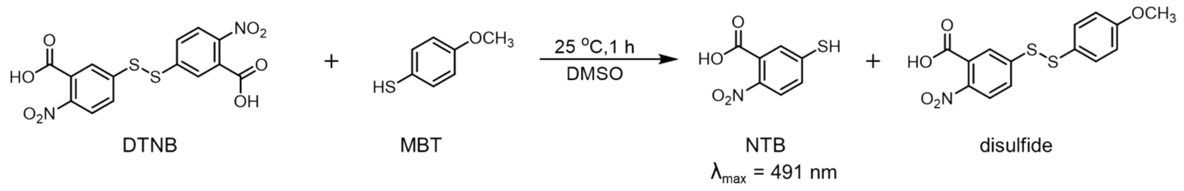

Figure S20. Model reaction for thiol−disulfide exchange using DTNB.

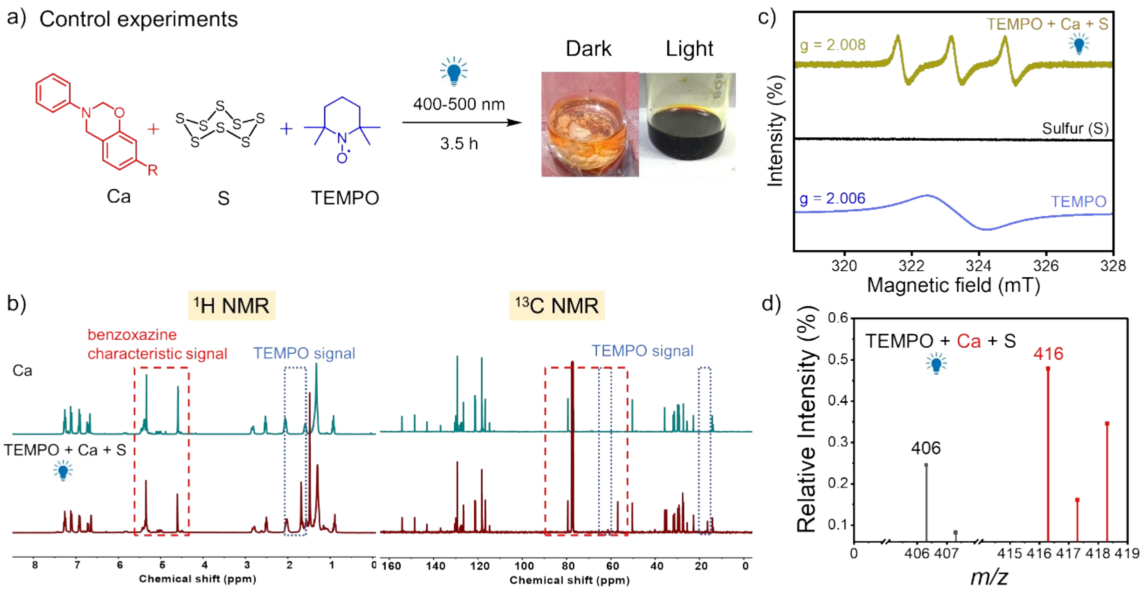

Figure S21. (a) Control experiments performed and corresponding digital images of samples. (b) ^{1}H and ^{13}C NMR of Ca and comonomer mixture in presence of TEMPO before and after irradiation. (c) EPR spectra of TEMPO, elemental sulfur and reaction mixture post 1 h irradiation; (d) Mass spectrum of reaction mixture after irradiation.

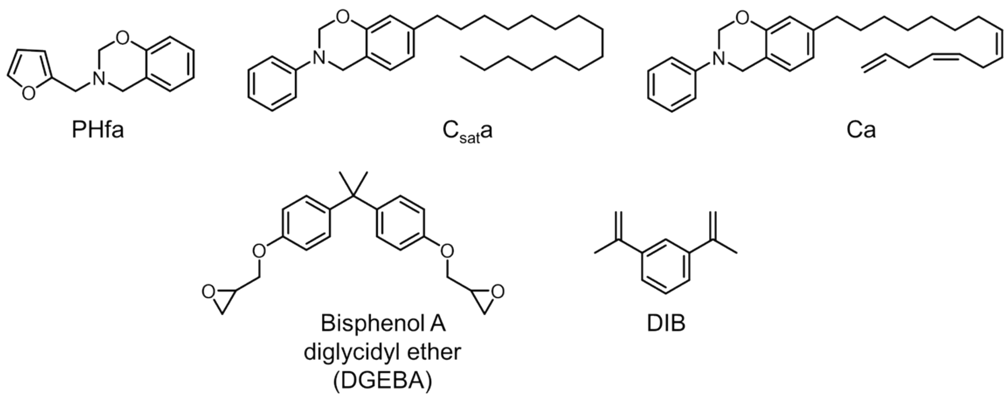

Figure S22. Different comonomers used to probe the photochemical reaction with elemental sulfur.

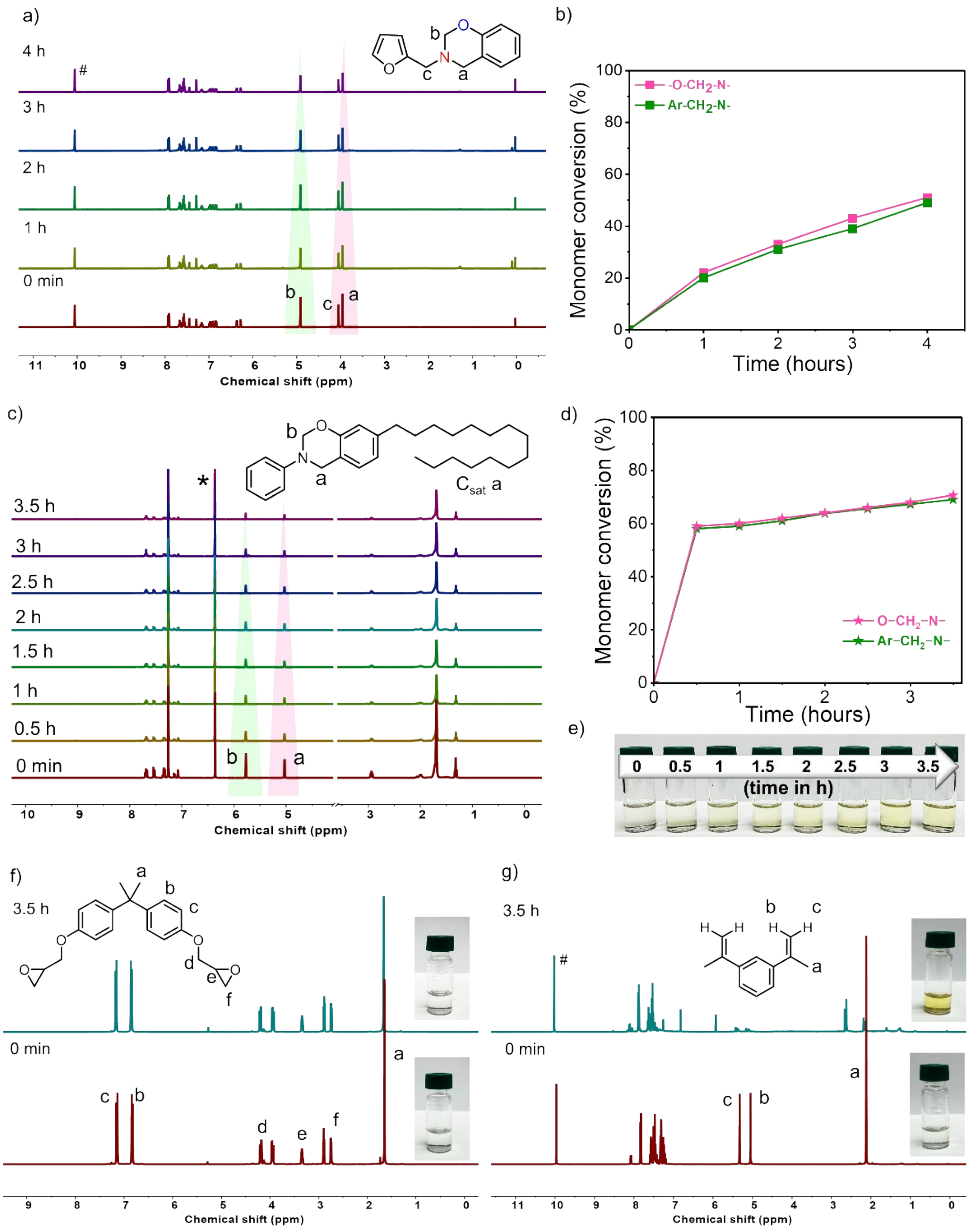

Figure S23. (a-d) NMR kinetics of photomediated reaction of elemental sulfur with PHfa and C_{sat}a was performed at 25 °C, and respective monomer conversion plots are presented following the the characteristic signal of Ar–CH_2–N and O–CH_2–N. Recorded in $CDCl_3$ at 400 MHz with internal standards ($^{\#}$/*).e) Digital images of the aliquots to monitor progress of reaction with time. Stacked ^{1}H NMR spectra of sulfur reaction with e) DGEBA and, f) 1,3-DIB at two different time.

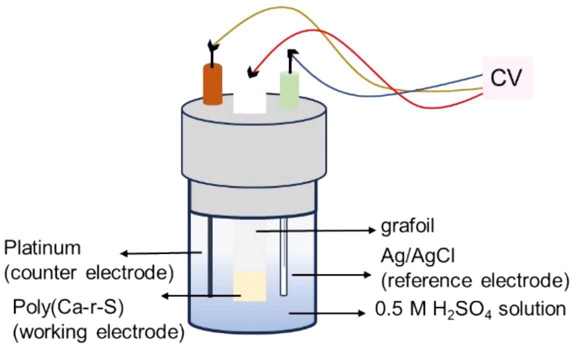

Figure S24. Electrochemical set-up for supercapacitor application.

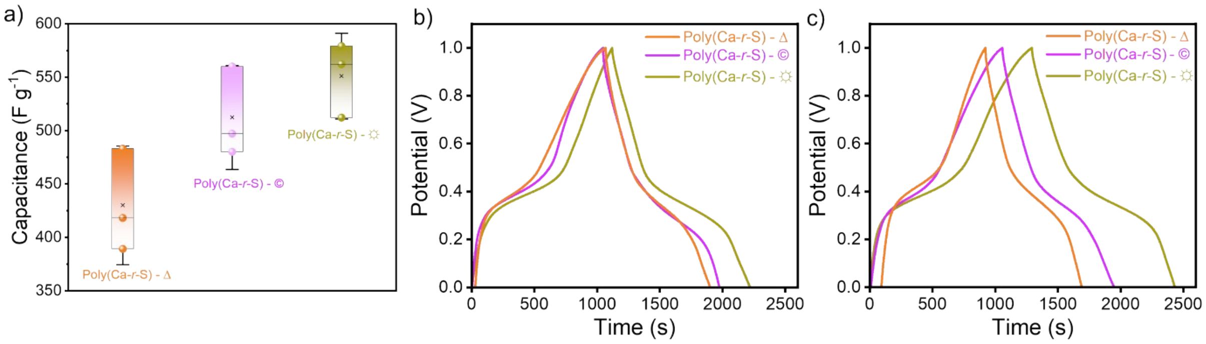

Figure S25. Electrochemical performance of poly(Ca-*r*-S) copolymers. (a) Specific capacitance determined from three different independent experiments (x: mean value with error bars). Respective (b-c) galvanostatic charge–discharge (GCD) profiles at 0.5 A g^{-1} for three independent experiments.

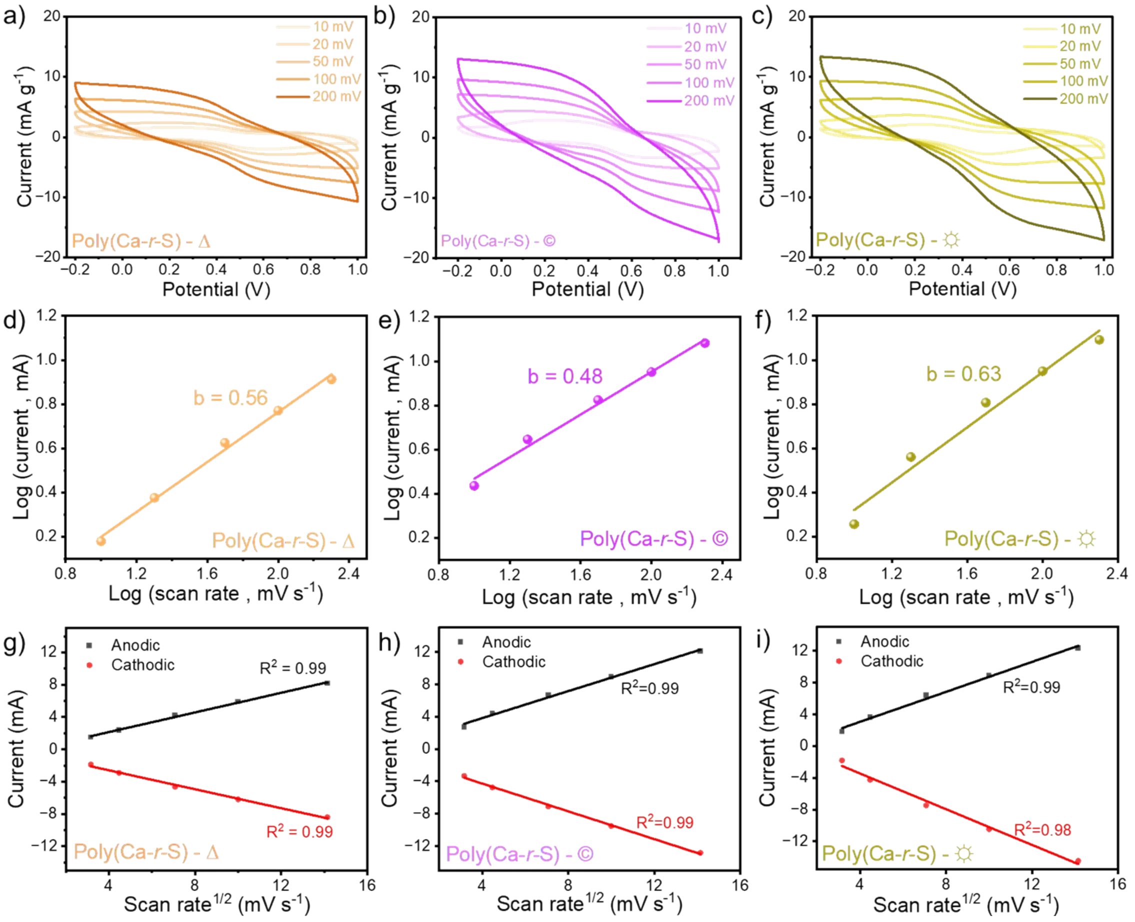

Figure S26. (a-c) CV curves, (d-f) determination of *b* value obtained by plotting log(*i*) vs. log(*v*), and (g-i) anodic and cathodic peaks current at various scan rates of all the synthesized copolymers.

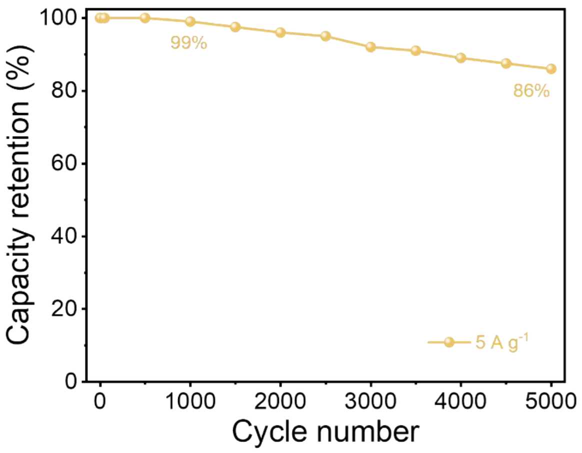

Figure S27. Cycling performance at 5 A g^{-1}.

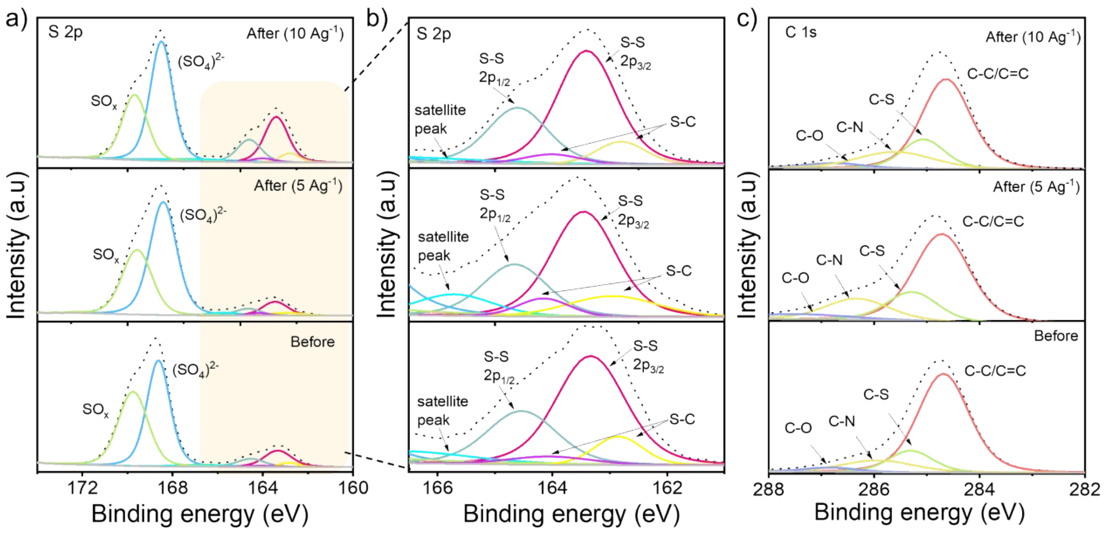

Figure S28. Comparison of XPS spectra. a) S 2p, b) C 1s, c) N 1s and, d) O 1s.

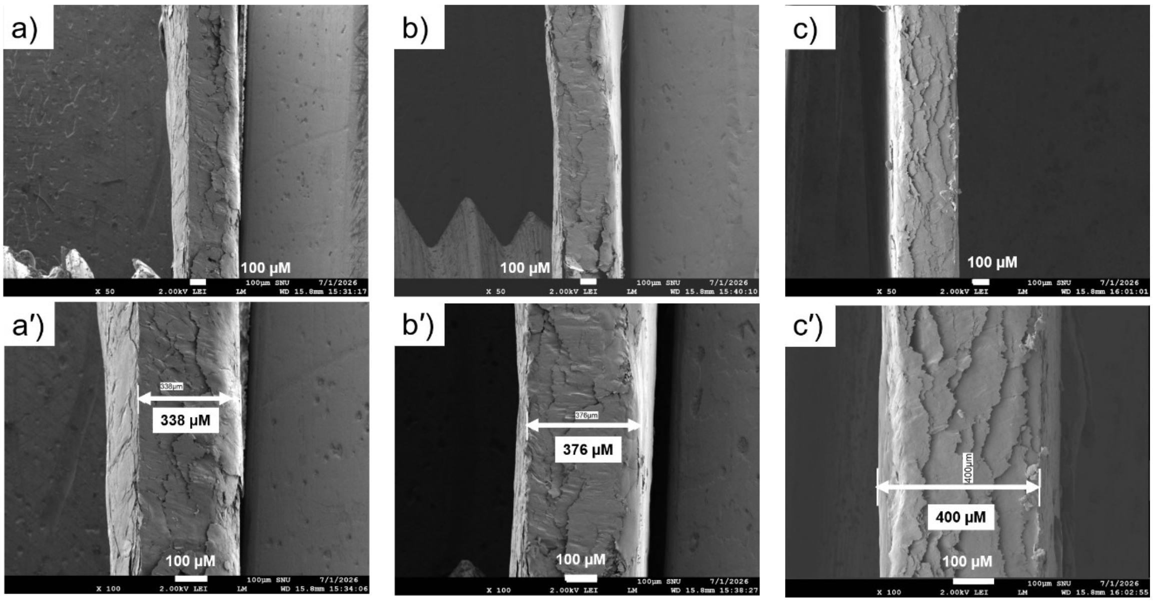

Figure S29. FESEM cross-section images of (a,a′) neat grafoil, and copolymer-coated grafoil electrode (b,b′) before cycling and (c,c′) after cycling.

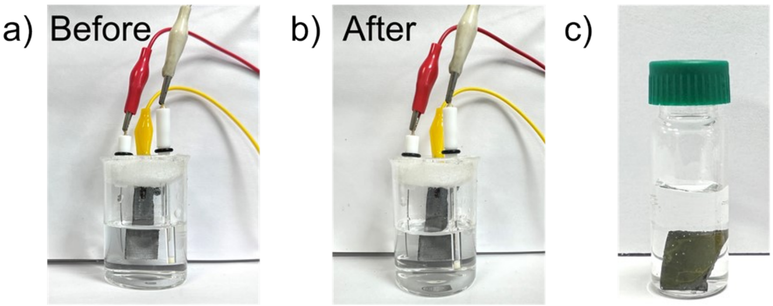

Figure S30. Digital images of the electrochemical analysis setup: (a) before cycling, (b) after cycling, and (c) stability of the electrode in 0.5 M H_2SO_4 after 30 days.

Table S9. Metal- and carbonization-free electrochemical characterization of polybenzoxazines as cathode material for supercapacitors.

**Material**	**Capacity ($F\ g^{-1}$)**	**Current density ($A\ g^{-1}$)**	**Cycle (retention %)**	**Electrolyte**	**Ref.**
TPA–DHTP–BZ POP	67.1	0.5	2000 (95.5%) at 5 A g^{-1}	1 M KOH	16
AC//PVA/PbzG5//AC	174.0	0.5†	5000 (76%) at 0.5 A g^{-1}	-	17
An-TPA POP	117	1†	10,000 (81.55 %)	1 M KOH	18
OVS-DHBZ POIP	58	0.5	2000 (95%) at 1 A g^{-1}	1 M KOH	19
NOSHPCs	216	0.5	5000 (90.38%) at 10 A g^{-1}	6 M KOH	20
TPEP-BZ	84	0.5	2000 (98.3%) at 10 A g^{-1}	1 M KOH	21
GO/NC nanocomposite	405.6	1	5000 (95.8%)	6 M KOH	22
Poly(Ca-*r*-S) - Δ	430 ± 13	0.5	-	0.5 M H_2SO_4	This work
Poly(Ca-*r*-S) - ©	512 ± 11				
Poly(Ca-*r*-S) - ☼	551 ± 9		5000 (75%) at 10 A g^{-1}		

†2 electrode setup

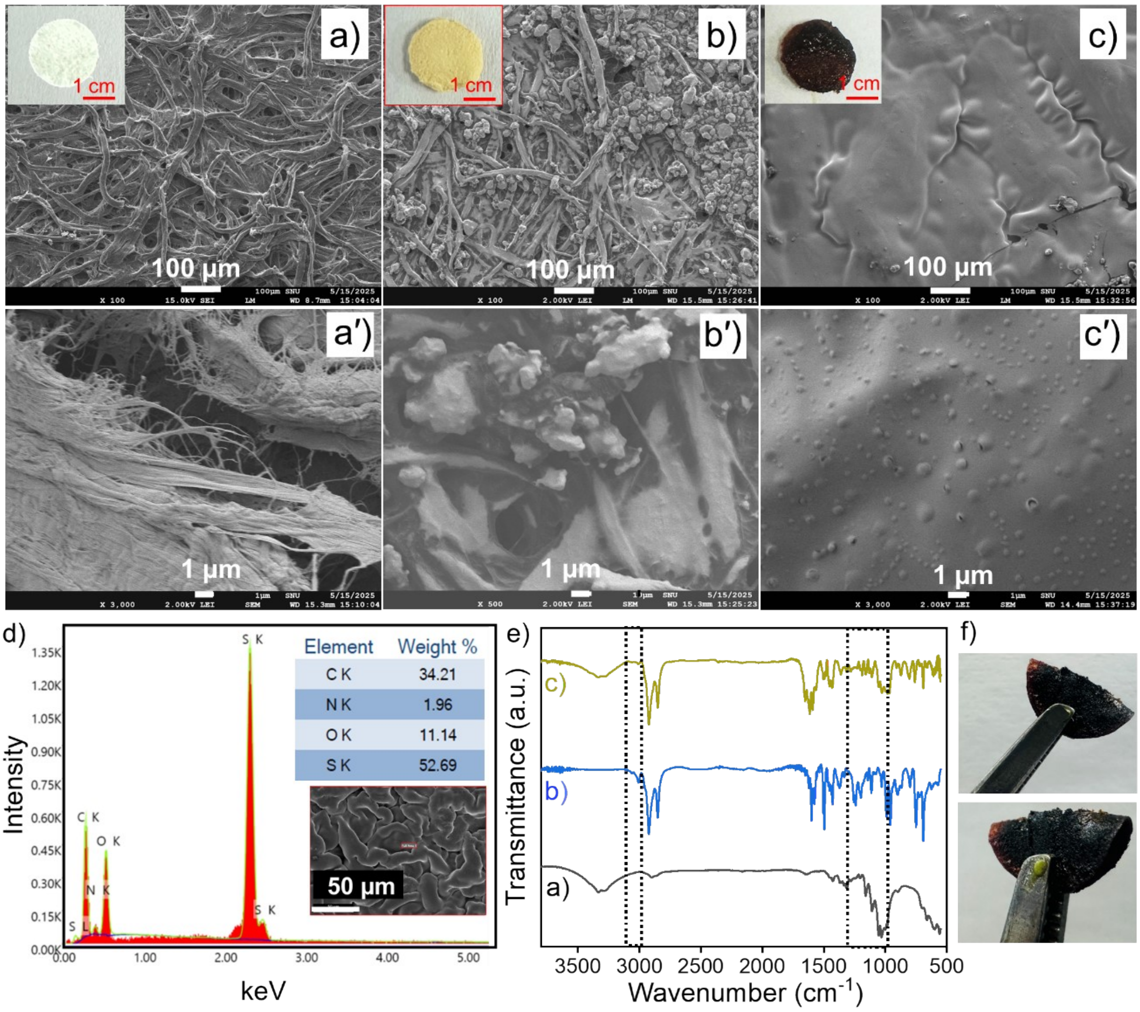

Figure S31. SEM images of (a, a′) filter paper, (b, b′) filter paper adsorbed with Ca and sulfur and, (c, c′) in-situ coated poly(Ca-*r*-S) on the filter post photo-irradiation, d) FTIR of filter paper, comonomer coated filter paper and post irradiation, e) EDAX (200 × 200 µm^2) showing the homogenous coating and f) optical images of copolymer coated filter paper.

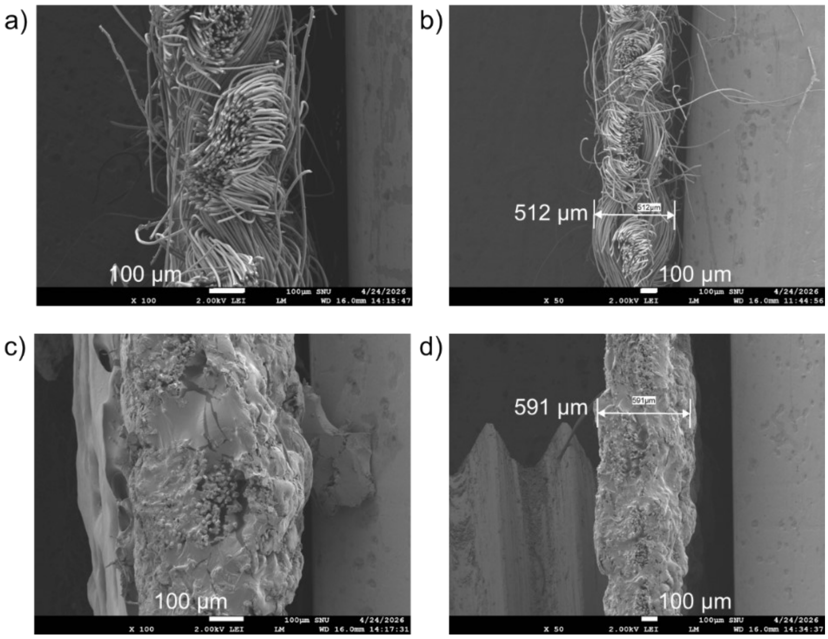

Figure S32. Cross sectional field-emission scanning electron microscope (FESEM) images of a-b) neat carbon cloth, c-d) coated carbon cloth at different magnifications.

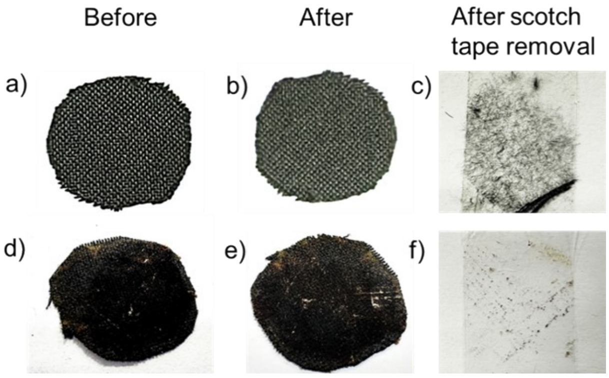

Figure S33. Cross hatch adhesion tests. Digital images of neat carbon cloth (a–c) and coated carbon cloth (d–f) before and after incision of the substrate, and scotch tape.

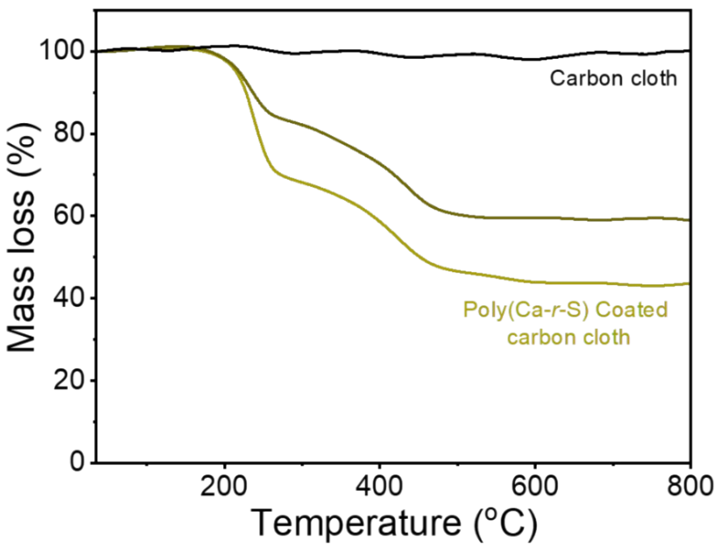

Figure S34. TGA of neat carbon cloth and poly(Ca-*r*-S) coated carbon cloth.

References:

1. S. Shukla, A. Mahata, B. Pathak and B. Lochab, *Cardanol benzoxazines–interplay of oxazine functionality (mono to tetra) and properties*. RSC Adv., 2015, **5**, 78071.
2. H. M. Smith and M. D. Pluth, *Advances and opportunities in H2S measurement in chemical biology*. JACS Au, 2023, **3**, 2677.
3. R. Schmidt, M. G. Logan, S. Patty, J. L. Ferracane, C. S. Pfeifer and A. J. Kendall, *Thiol quantification using colorimetric thiol–disulfide exchange in nonaqueous solvents*. ACS Omega, 2023, **8**, 9356.
4. S. Tape, *Standard test methods for measuring adhesion by tape test1*. ASTM International, Pennsylvania, United States, 2012.
5. M. Arslan, B. Kiskan and Y. Yagci, *Combining elemental sulfur with polybenzoxazines via inverse vulcanization*. Macromolecules, 2016, **49**, 767.

6. H. K. Lin and Y. L. Liu, *Sulfur radical transfer and coupling reaction to benzoxazine groups: A new reaction route for preparation of polymeric materials using elemental sulfur as a feedstock*. Macromol. Rapid Commun., 2018, **39**, 1700832.
7. M. Arslan, B. Kiskan and Y. Yagci, *Recycling and self-healing of polybenzoxazines with dynamic sulfide linkages*. Sci. Rep., 2017, **7**, 5207.
8. S. Sahu and B. Lochab, *Sustainable benzoxazine-sulfur copolymer with dynamic linkages: Recycling, reprocessing, self-healing, and shape recovery (R^2S^2)*. ACS Sustainable Chem. Eng., 2024, **12**, 7126.
9. O. Bayram, B. Kiskan, E. Demir, R. Demir-Cakan and Y. Yagci, *Advanced thermosets from sulfur and renewable benzoxazine and ionones via inverse vulcanization*. ACS Sustainable Chem. Eng., 2020, **8**, 9145.
10. S. Shukla, A. Ghosh, P. K. Roy, S. Mitra and B. Lochab, *Cardanol benzoxazines–A sustainable linker for elemental sulphur based copolymers via inverse vulcanisation*. Polymer, 2016, **99**, 349.
11. S. Sahu and B. Lochab, *Facile strategy for room temperature knitting of sulfur in polybenzoxazine: a new class of solution processable copolymers*. ACS Sustainable Chem. Eng., 2022, **10**, 12355.
12. W. J. Chung, J. J. Griebel, E. T. Kim, H. Yoon, A. G. Simmonds, H. J. Ji, P. T. Dirlam, R. S. Glass, J. J. Wie and N. A. Nguyen, *The use of elemental sulfur as an alternative feedstock for polymeric materials*. Nat. Chem., 2013, **5**, 518.
13. X. Wu, J. A. Smith, S. Petcher, B. Zhang, D. J. Parker, J. M. Griffin and T. Hasell, *Catalytic inverse vulcanization*. Nat. Commun., 2019, **10**, 647.
14. P. Yan, W. Zhao, F. McBride, D. Cai, J. Dale, V. Hanna and T. Hasell, *Mechanochemical synthesis of inverse vulcanized polymers*. Nat. Commun., 2022, **13**, 4824.

15. J. Jia, J. Liu, Z.-Q. Wang, T. Liu, P. Yan, X.-Q. Gong, C. Zhao, L. Chen, C. Miao, W. Zhao, S. Cai, X.-C. Wang, A. I. Cooper, X. Wu, T. Hasell and Z.-J. Quan, *Photoinduced inverse vulcanization*. Nat. Chem., 2022, **14**, 1249.

16. M. Ejaz, M. G. Mohamed and S.-W. Kuo, *Solid state chemical transformation provides a fully benzoxazine-linked porous organic polymer displaying enhanced CO_2 capture and supercapacitor performance*. Polym. Chem., 2023, **14**, 2494.

17. S. P. Asrafali, T. Periyasamy, G. A. R. Bari and S.-C. Kim, *Flexible composite hydrogels based on polybenzoxazine for supercapacitor applications*. Gels., 2024, **10**, 197.

18. A. F. Saber, S. Abdelnaser, A. F. EL-Mahdy and S.-W. Kuo, *One-pot synthesis of heteroatom-rich anthraquinone-based benzoxazine-linked porous organic polymers for high performance supercapacitors*. Electrochim. Acta., 2025, **511**, 145397.

19. M. Ejaz, M. G. Mohamed and S.-W. Kuo, *Benzoxazine-linked polyhedral oligomeric silsesquioxane: 3D porous organic-inorganic polymer for improved CO_2 capture and supercapacitor performance*. J. Taiwan Inst. Chem. Eng., 2025, 106098.

20. J. Jiang, M. Wang, W. Zhao, H. Liu, Y. Wang, P. Song and Z. Wang, *Hierarchical porous carbon materials derived from N, O, S-Containing bio-based polybenzoxazine for supercapacitors*. Eur. Polym. J., 2023, **191**, 112054.

21. M. M. Samy, M. G. Mohamed and S.-W. Kuo, *Pyrene-functionalized tetraphenylethylene polybenzoxazine for dispersing single-walled carbon nanotubes and energy storage*. Compos. Sci. Technol., 2020, **199**, 108360.

22. L. Wan, C. Du and S. Yang, *Synthesis of graphene oxide/polybenzoxazine-based nitrogen-containing porous carbon nanocomposite for enhanced supercapacitor properties*. Electrochim. Acta., 2017, **251**, 12.
